# Endoscope Capsules: The Present Situation and Future Outlooks

**DOI:** 10.3390/bioengineering10121347

**Published:** 2023-11-23

**Authors:** Rodrigo Gounella, Talita Conte Granado, Oswaldo Hideo Ando Junior, Daniel Luís Luporini, Mario Gazziro, João Paulo Carmo

**Affiliations:** 1 Group of Metamaterials Microwaves and Optics (GMeta), Department of Electrical Engineering (SEL), University of São Paulo (USP), Avenida Trabalhador São-Carlense, Nr. 400, Parque Industrial Arnold Schimidt, São Carlos 13566-590, Brazil; talitaconte16@gmail.com (T.C.G.); jcarmo@sc.usp.br (J.P.C.); 2 Academic Unit of Cabo de Santo Agostinho (UACSA), Federal Rural University of Pernambuco (UFRPE), Cabo de Santo Agostinho 54518-430, Brazil; oswaldo.ando@ufrpe.br; 3 Clinica Endoscopia São Carlos, Rua Paulino Botelho de Abreu Sampaio, 958, Centro, São Carlos 13561-060, Brazil; luporinidl@gmail.com; 4 Information Engineering Group, Department of Engineering and Social Sciences (CECS), Federal University of ABC (UFABC), Av. dos Estados, 5001, Santo André 09210-580, Brazil; mario.gazziro@ufabc.edu.br

**Keywords:** capsule endoscopy, endomicroscopy, endoscopy, GI tract, NBI, optical filters, PDT

## Abstract

This paper presents new perspectives on photonic technologies for capsule endoscopy. It first presents a review of conventional endoscopy (upper endoscopy and colonoscopy), followed by capsule endoscopy (CE), as well as their techniques, advantages, and drawbacks. The technologies for CEs presented in this paper include integration with the existing endoscopic systems that are commercially available. Such technologies include narrow-band imaging (NBI), photodynamic therapy (PDT), confocal laser endomicroscopy (CLE), optical coherence tomography (OCT), and spectroscopy in order to improve the performance of the gastrointestinal (GI) tract examination. In the context of NBI, two optical filters were designed and fabricated for integration into endoscopic capsules, allowing for the visualization of light centered at the 415 nm and 540 nm wavelengths. These optical filters are based on the principle of Fabry-Perot and were made of thin films of titanium dioxide (TiO_2_) and silicon dioxide (SiO_2_). Moreover, strategies and solutions for the adaptation of ECs for PDT are also discussed.

## 1. Introduction

Since there is no single method for correctly identifying all types of pathologies of the gastrointestinal tract (some methods are better at detecting tumors while others are better at detecting bleeding, etc.) we need to combine several techniques in order to achieve the best results using capsule endoscopy technology. This means that this paper is organized in sections to describe some of these start-of-the-art techniques.

### 1.1. Organization of the Paper

After an introductory section explaining the origins and advent of endoscopic capsules, the paper presents the benefits, contraindications, and drawbacks of its utilization. Next, its use in the main pathologies that affect the gastrointestinal tract is presented, and some of the commercial devices for capturing images and videos elucidating their technological mechanisms of scientific operation are presented in detail.

### 1.2. Capsule Endoscopy Origins

Due to the rapid development of medical devices, procedures that were previously invasive and causing discomfort became painless and almost imperceptible to the patients. In this context, so-called “medical images” are intended to provide clinicians with the ability to internally view the human body noninvasively and make the diagnosis more accurate and secure [1,2,3,4]. To reach this level, optical techniques have been very important for researchers and technology developers, because they are based on the analyses of light as a result of its interaction with matter, e.g., reflection, transmission, absorption, refraction, and diffraction [5,6,7,8,9].

**Figure 1:**
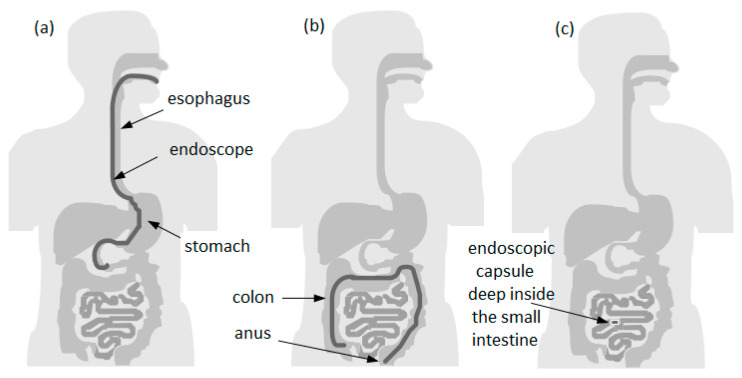
Comparison between covered areas of the diagnostic with endoscopy and endoscopic capsule: (**a**) upper endoscopy; (**b**) lower endoscopy; (**c**) area that cannot be reached by conventional upper endoscopy or even by colonoscopy.

In most cases, to perform the observation, diagnosis, and sometimes even treatment of pathologies in the gastrointestinal (GI) tract, a technique called endoscopy is used. The endoscopy of the GI tract is divided into upper endoscopy (or esophagogastroduodenoscopy, EGD) [10] and lower endoscopy (or colonoscopy) [11]. Figure 1a,b illustrate two types of endoscopic procedures, upper and lower, respectively. It can be observed that there are still some areas where conventional endoscopy is not able to reach. This restriction limits the possibility of detecting common anomalies like bleeding, ulcers, and tumors while they are in the early stages. In this way, the opportunity to control or even cure diseases before the need for complex treatments is lost [12].

Since the absence of accurate diagnosis often results in the degradation of symptoms and the rapid development of a disease, in order to improve diagnostics, a group of researchers from Baltimore invented the concept of a wireless endoscopic capsule in 1989 [13]. Later, in 2000, this concept was introduced by Given Imaging Inc., Duluth, GA, USA [14]. This technology brings to mind the futuristic concept in the 1966 movie “Fantastic Voyage”, where a submarine along with its crew is reduced to a microscopic size and injected into the bloodstream of a terminal patient to travel into a specific part of its body to destroy a malignant tumor with a LASER gun. The technology of wireless endoscopic capsules has revolutionized gastroenterology and simultaneously promoted a new diagnostic perspective on the GI tract that previously could only be achieved by surgery, such as certain portions of the small intestine [14]. Figure 1c shows an endoscopic capsule exam in the small intestine, which cannot be accessed with conventional endoscopy in most cases.

This paper focuses on the role of new endoscopic imaging technologies that emerged from the need for better visualization of gastrointestinal tract mucosa. These improvements are expected to provide more precise information for endoscopists, surgeons, physicians, and radiologists.

## 2. Capsule Endoscopy

### 2.1. Benefits of Capsule Endoscopy

There are multiple benefits offered by CE. First, the patients do not need sedation to undergo a CE analysis. The CE can analyze the entire GI tract from the esophagus, passing through the stomach, until the small intestine, which could not be properly analyzed through conventional endoscopy. The capsule has the size of a conventional vitamin capsule, and it can be easily swallowed, moving naturally through the GI tract until excretion. This fact indicates a painless procedure compared with the discomfort suffered by the long endoscopy sessions [15].

### 2.2. Drawbacks of Capsule Endoscopy

Despite all the benefits that a capsule endoscopy may offer, the drawbacks must also be considered. These disadvantages are divided into technical and physiological. The first group is related to the capsule itself, and the other one is related to the human body, or to be more specific, to the gastrointestinal system.

Sussman and Kulkarni gathered data from different perspectives about the risks encountered during capsule endoscopy [16]. The primary topics discussed in this study included capsule retention [17], patency [18,19,20], difficulty swallowing and aspiration [17,21], incomplete examination and suboptimal results [17,22,23], pediatric capsule endoscopy [24], and bowel preparation [17,25,26,27]. Among the technical complications, the most common problems were the presence of gaps in the recording, short duration or malfunction of the battery, failure in the capsule activation, and the inability to download the images [17].

Moreover, Penazzio’s study concluded that the endoscopic capsule could not be used to obtain biopsy specimens or for endoscopic treatment and could not be controlled remotely either [28]. Therefore, the risks of capsule endoscopy should be carefully reviewed, since every patient must be informed about possible complications that might arise after capsule examination.

### 2.3. Indications of the Capsule Endoscopy

The use indication of a capsule endoscopy is mainly for small bowel disease evaluation, due to the difficulty of diagnosis in this specific area. According to Jain [29], the indications are divided into two subsections: small bowel and esophagus.

Related to small bowel analysis, the indications are: obscure gastrointestinal (GI) bleeding, occult bleeding (positive FOBT), evaluation of iron deficiency anemia, Crohn’s disease, indeterminate colitis, assessment of mucosal healing, abdominal pain, graft-versus-host disease, surveillance of polyposis syndromes, celiac disease, suspected small bowel tumors, follow-up of small bowel tumors, follow-up of small intestine transplantation, evaluation of abnormal small bowel imaging, and evaluation of drug-induced injury.

Related to esophagus analysis, the indications are: Barrett’s esophagus (BE) and esophagitis and variceal evaluation. The main indications of each type of capsule are described in the following section. We note, however, that indications for esophagus analysis are a matter of ongoing research studies.

### 2.4. Contraindications of Capsule Endoscopy

Capsule endoscopy contraindications have been divided into groups to better understand safety issues. The two types of contraindications are the absolute and the relative ones. Absolute use contraindications include bowel obstruction, extensive and active Crohn’s disease, fistulas and strictures, intestinal pseudo-obstruction, and young children [29]; relative contraindications are for patients that have dysphagia, previous abdominal surgery, pregnancy, diverticulosis, cardiac pacemakers, and implanted electro-medical devices [29]. Suspected esophageal injuries or traumatic ruptures, hiatus hernias, and any follow-up for swallowed corpus alienum are also contraindications. The last two contraindications have been excluded from the absolute group, since some studies noted no interference of capsule endoscopy in the functioning of implantable devices [30,31].

### 2.5. The Problem of Capsule Mobility

Up to now, it is undeniable that an EC is a major comprehensive device for physiological measurements, with imaging and optical biopsy, as well as immunologic cancer recognition [6]. Unfortunately, the available ECs are passive, meaning that it is impossible to control their locomotion and steering for obtaining a better illumination and a higher rate of images within a selected mucosa spot [32]. New approaches have been researched in order to allow for the inclusion of such functions in ECs. In these contexts, two main approaches are candidates for locomotion control: internal and external. The internal approaches lead to on-board actuators such as vibratory actuators for self-propulsion [33], pedundulatory locomotion [34], legged locomotion [35], or even a swimming capsule with propellers [36]. These approaches reduce the tissue friction or push aside tissue around the capsule to pass through a collapsed bowel of fold. Nevertheless, these approaches reduce the EC’s life time and enlarge the size far beyond what is the standard (e.g., 26 mm in length and 11 mm in diameter) [37]. Moreover, the vibration approach causes effects on the capsule vision [38], whereas the legged approach could lead to the perforation of tissue or, even worse, to a stuck position where a surgical intervention is required for capsule removal. Additionally, these approaches need a considerable space for locomotion at the bowel wall. Alternatively, the external approaches focus on a magnetic field established between the EC and an external magnet controlled by an operator. Moreover, magnetic actuation is a very important trend in future applications of ECs [39,40]. The magnetic approach does not have mechanical parts, and therefore, it is not limited through the batteries, and at the same time, it does not cause effects on capsule vision. An elastic magnetic bracelet for attachment into ECs by covering its central portion was developed by Carpi et al. [41] and allows the user to steer the capsule according to the operator needs. However, this solution increases the total diameter of the EC, and it can result in an increased risk for the patient, because the magnetic bracelet can release from the EC during an exam. One alternative active magnetic platform for guiding endoscopic capsules (ECs) inside the human body was developed by Silva et al. [42] using two permanent magnets of neodymium (Nd-Fe-B) with a cylindrical shape and with a magnetization level of N48. One of the permanent magnets is external, while the other is internal to the human body. The external magnet provides a maximum magnetic field of 1.5 T and has a diameter of 11 mm and a length of 26 mm. The external magnet interacts with a small magnet placed inside the unit of an EC with a diameter of 3 mm and a length of 8 mm. The mobility tests of their EC concept were successfully validated through ex vivo clinical tests in porcine intestine. Another magnetic approach was developed by Keller et al. [43] and is based on a modified MRI system to control the navigation inside a water-filled stomach. This application provides 10 functions for basic capsule movements. The maneuverability of the EC is achieved by a complex set of electromagnetic coils (from the MRI systems). This set contains 12 coils for obtaining 5 + 1 degrees of freedom (DOF). This set of DOFs allows for magnetic steering in five degrees and an additional one to turn the patient [43]. Finally, the work by Tai et al. [44] resulted in a complex system of coils for providing magnetic levitation of the capsule by way of electromagnetic forces and, thus, for facilitating and controlling its motion.

## 3. Indications of Esophageal Capsules

### 3.1. Screening for Barrett’s Esophagus

Barrett’s esophagus consists of a metaplastic change of the esophageal mucosa’s lining, meaning that the columnar epithelium replaces the squamous epithelium that normally overlays the distal esophagus [45,46,47]. Barrett’s esophagus is an important risk factor for indication of esophageal adenocarcinoma, and several studies have indicated that its incidence has increased rapidly over the years [48,49]. The studies comparing the diagnostic yield between CE and conventional EGD demonstrated that CE was feasible, safe, and well tolerated by the patients. Moreover, the patients always preferred CE over unsedated EGD. On the other hand, the sensitivity of the esophageal capsule was variable between the detection of BE and another esophageal disease: 60–100% and 50–89%, respectively [50,51]. Although the results in terms of sensitivity are promising, studies have suggested that EGD is more cost-effective than CE for BE screening [52].

### 3.2. Screening for Esophageal Varices

The use of CE for detecting esophageal varices is not well defined due to the fact that all the studies present considerable heterogeneity between their findings.

Pena et al. found that an esophageal capsule could be used in the assessment of esophageal varices (EV). The sensitivity calculated in this study was 68.4% in detecting EV using CE against 95% using EGD. However, due to the minimal discomfort, lack of sedation, and low risk offered by CE, this technology is a possible substitute for EGD [53].

Groce’s study showed a sensitivity of CE in detecting EV around 78% and that CE may be superior to EGD for identification of small EV [54]. On the other hand, Einsen’s and Smith’s study indicated a better perspective on CE tests, with sensitivity reaching up to 100% [55,56]. The same results were found in Ragunath’s study [57]. The lowest sensitivity was showed in Jensen’s study, where it was only 8.3%, with modest accuracy of the CE in the identification of EV [58].

## 4. Indications of Intestinal Capsules

### 4.1. Intestinal Tumors

Tumors found in the small bowel (SB) represent 5% of all GI tract tumors and 2% of the cancer rates, despite a very low accuracy of the estimative, since the current methodologies have been proven to be inadequate [59]. On the other hand, the investigation of small bowel tumors with CE, an effective diagnostic modality, was established in 2004, and 8.9% of the patients who underwent the procedure were diagnosed with SB tumors. The expectation of the clinicians was that CE may lead to earlier detection and treatment of SB tumors, thereby improving the results for patients with neoplasms [60].

### 4.2. Obscure GI Bleeding

**Figure 2:**
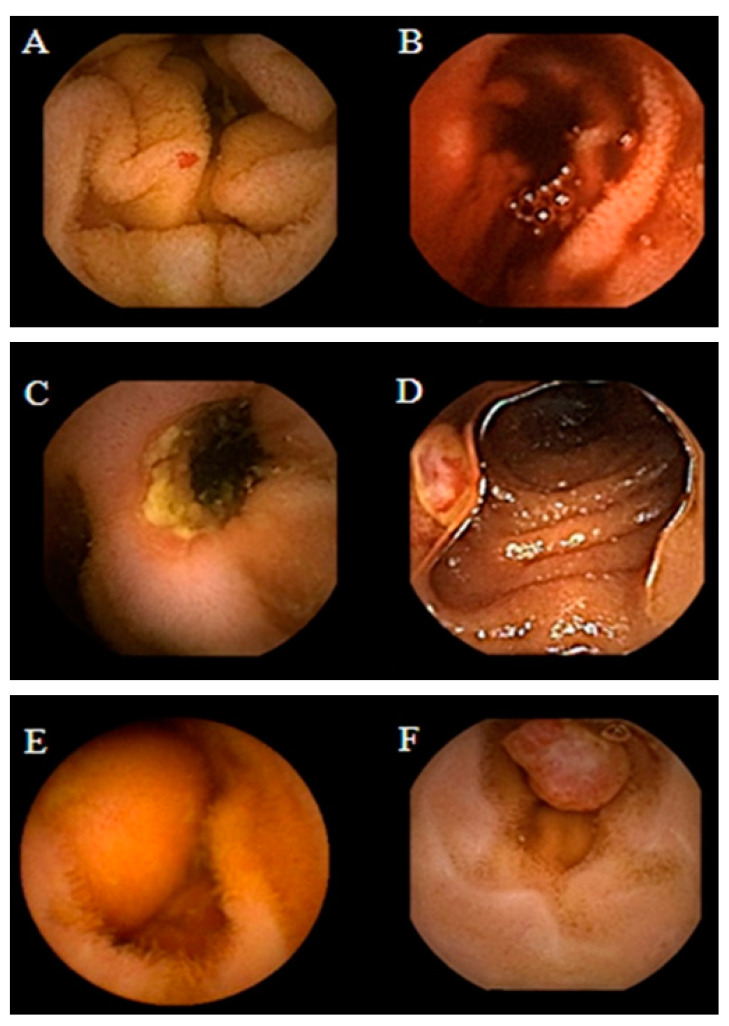
Spectrum of small bowel findings identified via capsule endoscopy (CE) in patients with obscure gastrointestinal bleeding (OGIB). (**A**) Nonbleeding angioectasia, (**B**) active bleeding, (**C**) ulcer with stenosis, (**D**) small bowel erosion, (**E**) submucosal mass, and (**F**) polyp [67].

Obscure GI bleeding can be defined by episodes of digestive bleeding, a positive fecal occult blood test, or chronic iron-deficiency anemia [61,62]. The complexity of GI bleeding relates to the fact that the bleeding can occur from multiple lesions at many sites in the GI tract. This pathology is evident to the patient but can be from a source which is not easily identifiable via conventional upper or lower endoscopy [63]. For that reason, and based on meta-analysis studies, the diagnostic yield of intestinal capsules ranges from 55% to 81%, which confirmed the superiority of CE diagnosis against other modalities of conventional endoscopy [64,65,66]. Figure 2 shows the small bowel findings of obscure GI bleeding achieved with intestinal CE.

### 4.3. Crohn’s Disease

Crohn’s disease is an inflammatory disease of the GI tract and often spreads deep into the layers of affected tissue. It can occur from the mouth to anus [68], although the probability of its incidence in the small bowel ranges from 30% to 40% of cases [69,70].

Generally, in order to identify an occurrence of Crohn’s disease, the clinicians must rely on a combination of clinical, endoscopic, and histological findings, because there is no single test that can fully diagnose the disease. Imaging studies normally lack sensitivity to be able to identify early lesions [29].

Schulmann et al. tested capsule endoscopy for the purpose of finding evidence of Crohn’s disease, and at that time, they agreed that full visualization and imaging of the entire length of the SB was unsatisfactory, and CE was considered a promising new approach for the diagnosis of SB diseases [71].

**Figure 3:**
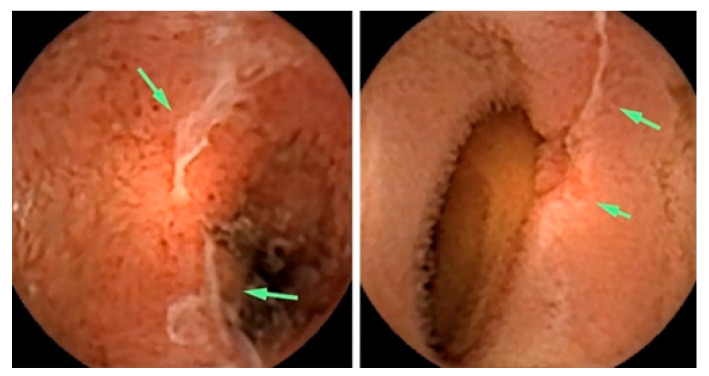
Active Crohn’s disease in the more proximal small bowel (SB), detected via CE. Images shown are ulcers detected via small bowel CE in the proximal SB. The green arrows indicate the ulcers [74].

Albert et al. compared CE with magnetic resonance imaging (MRI) and found that CE can detect limited mucosal lesions that may be missed by MRI and was slightly more sensitive than MRI: 12 versus 10 of 13 in suspected Crohn’s disease and 13 versus 11 of 14 in established Crohn’s disease [72]. Based on the stated facts, CE proved to be a good complementary method for diagnosing SB Crohn’s disease. More recently, Sange et al. state that CE innovation reduced reading time, improved diagnostic accuracy, and enhanced image quality [73]. Figure 3 presents findings of active Chron’s disease detected via intestinal CE.

### 4.4. Celiac Disease

The use of capsule endoscopy in patients with celiac disease consists of finding complications such as unexplained diarrhea, abdominal pain, and small bowel tumors. Fry et al. found a low yield for capsule endoscopy in patients with abdominal pain or diarrhea, and they recommended this type of evaluation as a first-line test. Moreover, the results showed a yield of 6% for abdominal pain, 14% for diarrhea, and 13% for both [75]. Based on the experiments conducted by [76,77,78,79] and presented at the ICCE consensus, for the diagnosis of Celiac disease with CE, Cellier et al. considered that there was enough evidence to support the use of CE in patients who have been treated and previously confirmed to have celiac disease [80].

### 4.5. Genetic Disorders

Soares et al. performed a study about Peutz-Jeghers syndrome (PJs)—an inherited gastrointestinal polyposis disorder, most commonly found in the small intestine. They found that CE offered excellent visualization of the small intestine and correctly identified all the patients having large polyps, although it missed 20% of the total number of them [81].

## 5. Commercial Capsules

Since the early years of conventional endoscopic procedures, the small bowel has been considered technically difficult to examine due to its length and location. The concept of a capsule indicated for small bowel analysis was developed by two groups. In 1996, a gastroenterologist named Dr. Paul Swain demonstrated the first live transmissions of a capsule analysis using a pig’s stomach. In 1997, he decided to collaborate with Dr. Gavriell Iddan, a mechanical engineer [14,82,83]. In 2000, they published successful animal trials [82], and in 2001, they published human studies on the use of capsule endoscopy in clinical trials [14,84]. At this time, the Food and Drug Administration (FDA) approved the capsule endoscopy [62].

Previously, the small bowel was a difficult organ to explore with the available technologies, such as conventional endoscopy or radiological and nuclear techniques, due to anatomical or physiological causes. In 2005, the role of the capsule endoscopy was widely discussed at the International Conference on Capsule Endoscopy (ICCE) [81,85,86,87,88,89,90], a symposium organized and sponsored by Given Imaging.

Nowadays, there are several brands of capsules which are approved by the FDA, like PillCam by Given Imaging, OMOM by Jinshan Science & Technology, EndoCapsule by Olympus, and MiroCam by Intromedic (Seoul, Republic of Korea). Next, we describe each capsule, its advantages, drawbacks, and main components.

The approval from the FDA for the PillCam SB capsule was received in 2003, and the market release was indicated for use in pediatric patients, aiming specifically at the diagnosis of pathologies of the small bowel [65,91].

The PillCam capsules produced by Given Imaging Inc., Duluth, GA, USA, are divided into three categories, small bowel (SB), esophagus (ESO), and colon (COLON), which have video cameras designed for imaging the gastrointestinal tract. Each of them is equipped with a battery, LEDs (light-emitting diodes), and a transmitter with an antenna. All these components are enwrapped in a biocompatible plastic casing, and the capsule size is about 26.4 mm length and 11.4 mm diameter [92].

The PillCam SB category is subdivided into SB, SB2, and SB3. PillCam SB capsules incorporate one video camera. PillCam SB2 consists of a fixed-frame-rate second-generation capsule, and the PillCam SB3 has enhanced imaging capabilities with an adaptive frame rate (AFR) [92]. These capsules are indicated for the monitoring of lesions that may show Crohn’s disease that is not detected through upper and lower endoscopy. They are not indicated for patients with GI obstruction, strictures, or fistulas, patients with cardiac pacemakers or any implanted electro-medical devices, and for patients with dysphagia or other swallowing disorders.

The PillCam ESO category has two variants—PillCam ESO 2 with a fixed high-frame rate and PillCam ESO 3 with a fixed high-frame rate and enhanced imaging capabilities [92]. The PillCam ESO capsules are composed of two video cameras, and they can be used for the investigation of esophageal disorders, such as esophageal varices, esophagitis, and Barret’s esophagus [93], and in patients complaining about heartburn [94,95].

The PillCam COLON capsules also contain two video cameras, and this category is divided into two variants—COLON 1 has a fixed-frame-rate capsule, while COLON 2 has an enhanced imaging capability, AFR- [92]. Both PillCam ESO and PillCam COLON are contraindicated for patients with the profile already described in the PillCam SB section. This capsule is indicated to investigate intestinal disorders and tumors.

After various studies showing the risks of capsule retention, Given Imaging Inc. created the Patency Capsule (PC) System, an ingestible, dissolvable, and disposable capsule, composed of biocompatible materials. The patency capsule is given before a video capsule endoscopy in order to prevent or minimize the risk of capsule retention [96]. The body of the capsule is composed of compressed lactose that dissolves in GI liquids and 5% barium sulfate, which makes the capsule radiopaque. The system is composed of a nonvideo disintegrating capsule, radiofrequency identification (RFID) tag, and an RFID scanner [97]. The PC is supposed to remain intact in the GI tract for about 80 h according to its design, and after that, if not excreted yet, it disintegrates spontaenously [98].

In 2005 Jinshan Science and Technology Company (Chongqing, China) released to the market the OMOM CE. This CE is indicated for investigating obscure gastrointestinal bleeding (OGIB), abdominal pain or diarrhea, partial intestinal obstruction, suspected inflammatory bowel disease, and tumors [99]. A study showed that the visualization of the entire small bowel was achieved in 75% of patients who had undergone the procedure with the OMOM. In the patients with suspected small bowel (SB) disease, the detection of abnormalities was 70.5%. The diagnostic yield for patients with OGIB was 85.7%, while the detection in cases of abdominal pain or diarrhea was around 53.3% [99].

Olympus Medical Systems (Olympus, Tokyo, Japan) received marketing clearance from the FDA in 2007 for their EndoCapsule endoscope system [100]. This capsule was compared with the PillCam SB and classified with the same quality level by Pennazio [28] et al. The EndoCapsule capsule contains a camera, a transmitter, batteries, and a light source. It differs from the PillCam capsule in that it has a high-resolution image chip and an external real-time viewer [101,102].

A prospective randomized comparison between both capsules—Given PillCam SB and Olympus EndoCapsule—was carried out by Hartmann et al. In this study, it was shown that the Olympus EndoCapsule could detect more GI bleeding sources than the Given PillCam SB, although the difference was only numerical and statistically nonsignificant [103].

The Korean MiroCam (Intromedic) is another capsule with similar components to the capsules from Given Imaging and Olympus. The first clinical trial using MiroCam was in 2009, involving 45 patients. The quality of the image was rated as good in 91.1% of the cases, and the transmission rates of the captured image in the stomach, small bowel, and colon were 99.5%, 99.6%, and 97.2%, respectively. The authors disclosed that MiRo was safe and effective for investigating the entire SB, offering a good image quality and real-time feasibility [101].

Moreover, a study carried out in 2012 showed that the evaluation of the entire SB using MiRoCam was achieved in 96% cases, and relevant lesion findings occurred in 58% of patients. They also considered MiRoCam a safe and effective tool for exploring SB with a high completion rate [104]. Table 1 summarizes the commercial capsules available, as well as the indications, imaging system, size, and respective references with studies about each type of capsule.

## 6. New Functionalities for Capsule Endoscopy

Although numerous results from clinical studies and experiments of endoscopic capsules are being presented, the unfeasibility of motion control of the capsule makes the diagnostic of the gastrointestinal tract insufficiently accurate. Since the impossibility of any motion control of the capsule has arisen, studies about possible solutions for motion control have been described in some patents [105,106,107,108].

The basic concept behind these patents were capsules being intrinsically controllable by including induction coils or magnetic parts inside an invented capsule structure in the interest of making it responsive to an external magnetic field. However, as this kind of solution required a characteristic design, like the structure of capsule, geometrical shape, and magnetic properties, the costs of these would be raised.

Moreover, a different solution by Carpi et al. [109] was disclosed in 2006, which on the contrary allowed for the control of a traditional and commercially available endoscopic capsule without any structural modification. Their solution proposes a technique that exploits magnetic shells to be applied on traditional capsules prior to their use [109].

**Figure 4:**
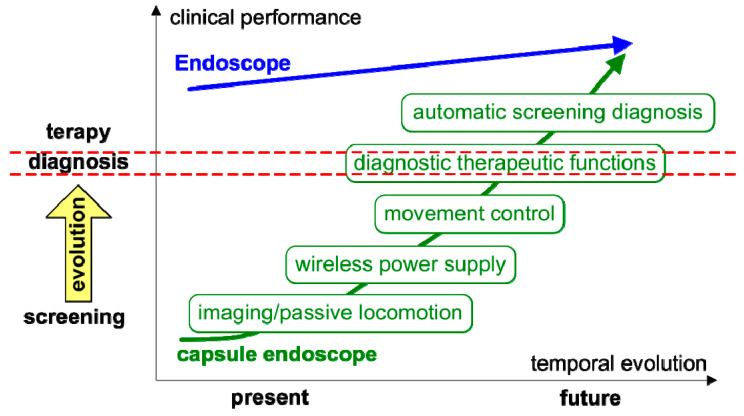
Roadmap for capsule endoscopy presented by Mr. Shimoyana, the President of Olympus, during the MicroMachine Summit 2005 (1–4 May 2005, Richardson, TX, USA) [110]. The red dotted mark the separation of diagnostic functions from therapy functions and simple visualization.

Figure 4 illustrates the personal vision of the President of Olympus, Mr. Shimoyana, during his talk at the MicroMachine Summit 2005 [110]. From his view, the capsule endoscopy would have a rapid growth compared with the conventional endoscopy. The reason for this increase might be because of all the functionalities that can be integrated into the endoscopic capsule. The growth rate is directly related to the number of possible functions that can be incorporated in the endoscopic capsule.

In this context, it is important to discuss the new technologies that can be integrated into the capsule endoscopy in order to increase the performance in screening, diagnosis, and therapy. In the following sections, we present the concept of confocal laser endomicroscopy, photodynamic therapy, narrow-band imaging, and how those technologies can be integrated into the endoscopic capsules.

## 7. Photodynamic Therapy

It is well known that light has been used as a therapy since the ancient civilizations, but until the last century, photodynamic therapy (PDT) was not yet developed. Since then, the applications of PDT have been tested by clinicians for use in oncology, such as in treatment of cancers of the neck, brain, breast, head, lung, pancreas, prostate, skin, intraperitoneal cavity, and gastrointestinal tract [111,112,113,114]. In this context, PDT represents an encouraging method for the treatment of cancer and even nonmalignant conditions [112].

The technique of PDT combines an administration of some photo-sensitizing therapies and an exposure of the tissue to visible light in the range of 400–760 nm. When light with an appropriate wavelength encounters the photosensitizer, the molecule is excited. This interaction generates a liberation of singlet oxygen, promoted by the series of molecular energy transfers. The singlet oxygen is a highly reactive and cytotoxic species and results in cell death [115].

The activation wavelength of light differs according to the site where we attempt to perform the therapy; usually wavelengths between 630 and 700 nm have been shown to lead to better results. As our focus in this review is the GI tract, the activation wavelengths for esophageal and gastric cancers are 630 nm and 635 nm [113,114].

PDT can be coupled to conventional endoscopes or even to the endoscopic capsules. Following the same logic of capsule endoscopy with NBI technology, we can also fabricate optical filters with a different wavelength, which can be used for PDT, and then integrate both technologies in the capsule. The problem of coupling PDT to the endoscopic capsules is that they must have integrated motion control in order to guarantee that the therapy is being delivered at the exact sites of interest.

Before we discuss the clinical application of using PDT as a therapy for diverse cancer types, we may first consider the treatment specificities and indications. Primarily, PDT is a local treatment, rather than systemic, and therefore, it is only suitable for localized disease. Secondly, when compared with other types of treatment, such as radiotherapy and chemotherapy, PDT represents a much faster and cost-effective treatment. Lastly, a huge advantage of PDT is that the limited light penetration protects healthy tissue beneath the tumor (or region of interest) from phototoxicity. Moreover, the treatment can be repeated in case of recurrence of the disease in the previously treated area [116].

Studies on PDT in the treatment of esophageal cancer were firstly carried out as a palliative for obstructive tumors; McCaughan et al. stated that the operative risk was minimal, and PDT had the ability to destroy the tumor as well as to increase the size of the esophageal lumen [117]. Schweitzer’s, Qumseya’s, and Moghissi’s studies confirmed the efficacy of PDT as a palliative therapy of dysphagia, evidencing the need for the development of more tumor-specific photosensitizers [118,119,120]. Nonetheless, side effects of PDT for esophageal cancer were listed in other studies, such as skin photosensitivity, stenosis, fistulas, and perforations (reported in up to 50% of the patients) [118,121,122].

PDT is also suitable for treatment of Barret’s esophagus. There is an estimate that shows that 50% of esophageal cancers develop from Barret’s esophagus. Therefore, effective treatment of Barret’s esophagus is very important [123]. PDT combined with long-term acid inhibition provided effective endoscopic therapy for elimination of Barrett’s mucosal dysplasia, superficial esophageal cancer, and also reduced/eliminated Barrett’s mucosa [124]. Later, the same group reported a conversion of approximately 80% of treated Barrett’s mucosa to normal squamous epithelium in all 100 patients who had participated in the study [125].

**Figure 5:**
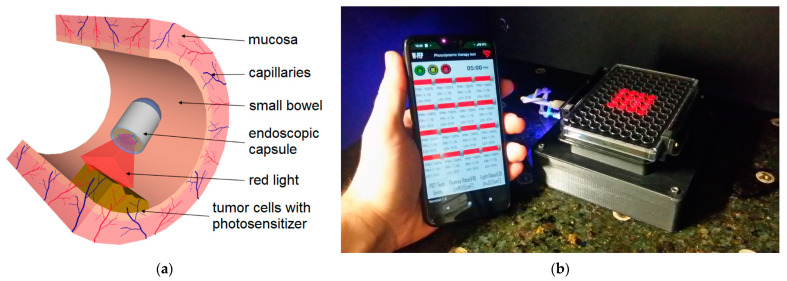
(**a**) An illustration of the concept of a compact PDT system to mount at the tip of an endoscopic capsule. (**b**) A photograph of a functional prototype of an evaluation system side-by-side and connected with a smartphone running a host application.

As shown in Figure 1, conventional endoscopy has the particularity and drawback of not being able to observe the entire small intestine, only a small portion of it. This is mainly due to the possibility of an increased risk of perforation of the intestine, taking into account its reduced thickness and sinuous structure [12]. On the basis of the above reasons, the endoscopic capsule (EC) becomes an excellent means of to implementing a form of photodynamic therapy (PDT) using light-emitting diodes (LEDs) with a wavelength in the region of red (e.g., from 620 nm to 750 nm) that can be incorporated into EC, as shown in Figure 5a [126], which allows this equipment the ability to perform treatment in the GI system. Naturally, it is necessary to carry out preliminary tests on biopsies of the patient’s own cells in order to define the doses of both the photosensitizers and light, as well as the exposure time in the endoscopic capsule to be applied during the treatment itself. The work developed by Gounella et al. [127] presents a platform for PDT, which validates such a procedure with the 5-aminolevulinic acid (ALA) photosensitizer on in vitro assays of human gastric adenocarcinoma cells. This work also presents other photosensitizers that are available commercially or in clinical tests and their main characteristics [127]. Figure 5b shows a functional prototype of an evaluation system side-by-side and connected with the smartphone running a host application.

## 8. Laser Endomicroscopy

Confocal laser endomicroscopy (CLE) is an imaging technique that uses a low-power laser to focus on a single point in a microscopic field of interest. The term confocal comes from the fact that the lens used in this technology allows for the illumination and detection systems to be aligned in the same focal plane [128,129].

**Figure 6:**
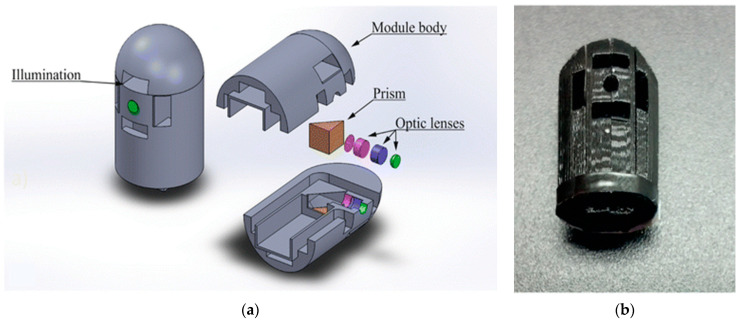
(**a**) CAD design of the lens holder. (**b**) Lens holder prototype obtained via 3D printing [126].

Costa et al. proposed an integration of an imaging magnification optical microsystem (IMON), including a PDMS lens, which was able to perform in vivo and real-time tissue microscopy (Figure 6). With a total length of 12.164 mm and a lateral lens assembly of 3.894 mm, a paraxial magnification of 4–14 times was achieved with great performance [126]. In this sequence of ideas, another similar IMON for ECs can be found in the work developed by Ribeiro et al. [130]. Such an IMON has a diameter of only 11.2 mm and a length of 18.6 mm and comprises an imaging system with a dedicated IMOM and light-emitting diodes (LEDs). Moreover, they fabricated and integrated microlenses that have been fabricated using the “hanging droplet” approach in the IMOM subsystem to provide an image magnification of 4×, with an improvement of 30% in the optical irradiance from the LED illumination [130].

**Figure 7:**
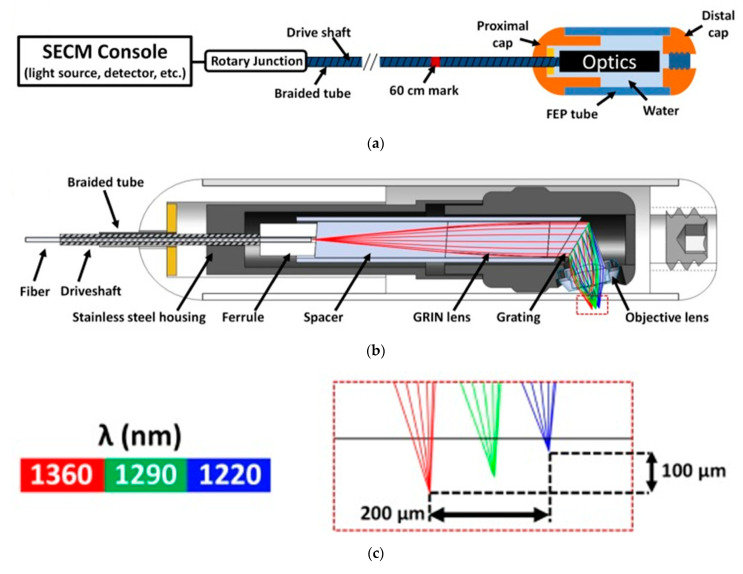
(**a**) Schematic view of SECM and the SECM tethered capsule. (**b**) Optical design (OD) integrated to the mechanical design of the capsule. (**c**) Close-up view of the tilted line based on the OD simulation [131].

Tabatabaei et al. reported on the development of a confocal microscopy capsule for diagnosis and monitoring of an esophageal disease called eosinophilic esophagitis (EoE). The EoE is an allergic condition characterized by eosinophils infiltration of the esophageal wall. Previously, the treatment offered for EoE required multiple follow-up sedated endoscopies and even biopsies to confirm the complete elimination of eosinophils. They developed a swallowable capsule which implements a high-speed fiber-based reflectance confocal microscopy, named spectrally encoded confocal microscopy (SECM). They presented imaging of esophageal biopsies from EoE patients ex vivo, demonstrating the ability of SECM to visualize individual eosinophils [131]. Figure 7 shows the schematic of the SECM clinical system.

As we can see from the presented studies, CLE integration in the CE is still a new field of study and is underdeveloped. Nevertheless, there are sufficient indications that this is a promising technology, and it can be expanded to the analysis of various diseases of the GI tract.

**Figure 8:**
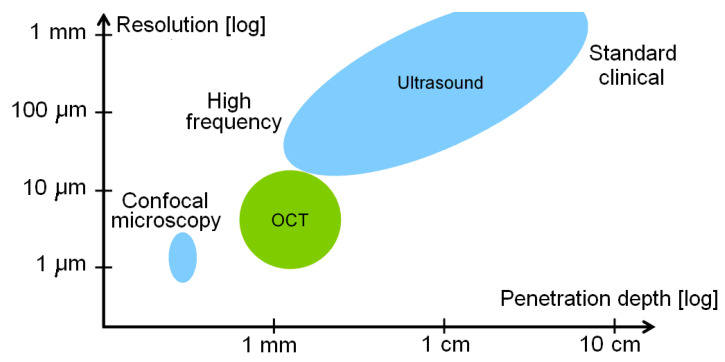
Comparison of resolution and imaging for ultrasound, OCT, and confocal microscopy.

An optical coherence tomography (OCT, also known as volumetric laser endomicroscopy) is a noninvasive optical diagnostic tool that enables in vivo cross-sectional tomographic 3D visualization of internal microstructures and functional information in biological systems. OCT is based on the measurement of reflected light from the tissue optical interfaces and uses the principles of optical interferometry, which is capable of imaging tissue at a micron-level resolution [132]. Another great advantage of OCT (see Figure 8) is a good compromise between the spatial resolution and penetration depth [133]. Typically, image resolutions of 1 μm to 15 μm can be achieved with OCT measurement, corresponding to one or two orders of magnitude higher than the ones with conventional ultrasonic scan. Confocal microscopy uses point illumination and a spatial pinhole to eliminate out-of-focus light in specimens to achieve a resolution of submicrometers [134]. However, the image penetration of confocal microscopy is limited to a few hundred micrometers in general scattering media, which is much lower than those penetrations achieved with OCT, which can penetrate 2–3 mm. OCT presents additional merits such as contactless measurement, relatively simple setup and computation, fast scan, and display.

**Figure 9:**
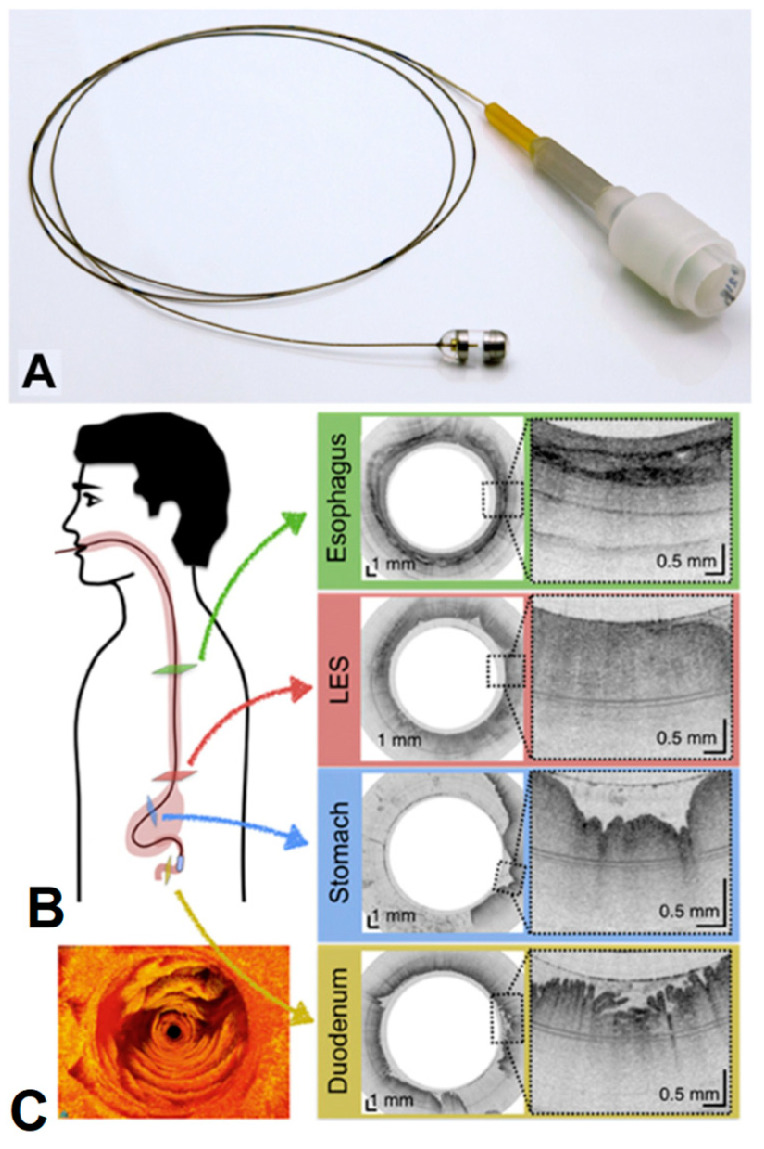
(**A**) Photograph of the TCE. (**B**) A representation of the procedure and TEC images of the esophagus (green), lower esophageal sphincter (red), stomach (blue), and duodenum (gold). (**C**) Three-dimensional reconstruction of optical coherence tomography-based tethered capsule endomicroscopy (TCE) images, view of the second portion of the duodenum from a 30-year-old male participant [135]. Dashed lines show zooms on selected regions of TCE images.

More recently, in 2018, the research group of G. J. Tearney [135] proposed a swallowed tethered capsule endomicroscopy (TCE) for microscopic imaging of the esophagus, stomach, and duodenum without sedation in humans by using a balloon catheter and OCT technology. The tethered capsule has a diameter ranging from 11 to 12.8 mm and a length of 24 to 24.8 mm. A 2 m long tether that connects it to an OCT imaging console allowed for the real-time acquisition of images at a frame rate of 20 fps, with an axial resolution (penetration depth) of 10 µm, and resolution of 35 µm along the lateral axis in two-dimensional cross-sections (Figure 9).

**Figure 10:**
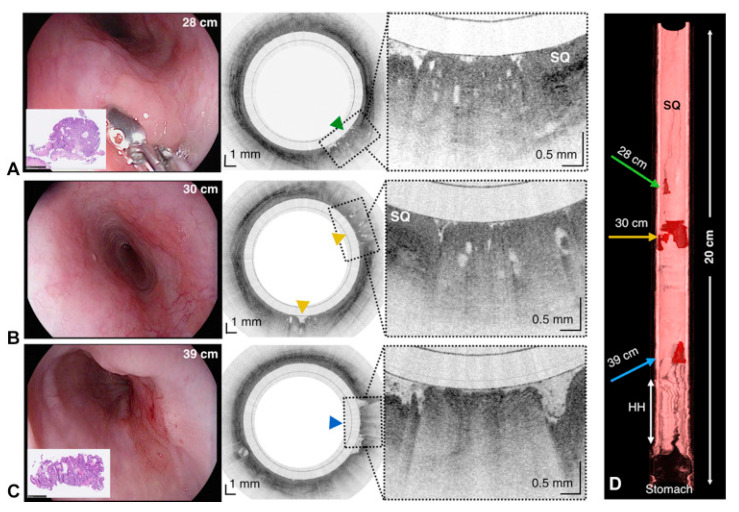
(**A**) Endoscopic photograph and corresponding TCE image of an 84-year-old man with a treated Barrett’s esophagus and intramucosal carcinoma; the lesion is shown at 28 cm by green arrowhead. (**B**) Endoscope and TCE images at 30 cm with 2 lesions (yellow arrowheads). (**C**) Photograph and TCE image at the gastroesophageal junction with significant architectural atypia, suggestive of cancer (blue arrowhead). (**D**) Three-dimensional rendering of the TCE dataset. SQ means Squamous epithelium, and HH means Hiatal hernia [135]. Dashed lines show zooms on selected regions of TCE images.

The capsule is reused by withdrawing it through the mouth and followed by a disinfection. In this work, the images acquired via OCT were compared with histopathology findings (when available), and the safety of the TCE was proven, making it feasible as a procedure for obtaining microscopic images of the upper gastrointestinal tract at a high resolution without endoscopic assistance and/or the need for sedation, as presented in Figure 10.

The ultimate aim of one day having ECs for OCT on the market is expected to be a reality; when analyzing recent works, a panoply of optical devices can be integrated at the wafer level in silicon in order to implement the required Michelson’s interferometer [136]. Other materials, which can include glass, are feasible candidates for heterogeneous integration using multichip module (MCM) techniques with CMOS imagers [137]. These kinds of microsystems are able to pave the way for having OCT in ECs in the near future.

## 9. Spectroscopy

It is of major importance that the detection of cancer at the dysplasia stage, e.g., before the occurrence of visible changes at a macroscopic level on the tissues. A dysplasia is not more than a precancerous change in the gastrointestinal tissue [138]. The early detection of dysplasia can increase the chance of successful treatment and full survival of the patient [138,139]. These kinds of changes are very difficult to identify and detect with conventional illumination and visual inspection, because a large area of changes in the tissue is required for it to become easily observable [139,140]. Spectroscopic techniques are based on the interactions between the light and the tissue. These techniques can be based on diffuse reflectance or based on fluorescence. Nonetheless, both have the potential to allow for the detection of small changes in the tissue, e.g., macroscopically invisible lesions on the tissue surface [140,141]. Moreover, some morphological and biochemical changes in the tissues (related to early cancer progression) can modify the shape and intensity of the signals that are involved in the diffuse reflected and fluorescence signals. The extraction of the diffuse reflectance signal of a tissue can be used to detect small changes related to cancer progression, since its intensity and shape are affected by absorption and scattering effects [142]. As a result, an increase in hemoglobin results in a reduction in the diffuse reflectance signal, because this optical effect is associated with angiogenesis during cancer progression. Moreover, as the dysplasia is progressing towards a cancer, the thickness of the epithelial tissue increases, thus reducing the quantity of light that reaches the deeper tissues. Therefore, a smaller quantity of light reaches the collagen fibers (the main tissue scatterer) in the connective tissue, decreasing scattering and, consequently, decreasing the diffuse reflectance signal intensity [140,141].

Several developments and research on systems for spectroscopy signals extraction and detection of gastrointestinal dysplasia can be found in the literature [143,144,145,146]. However, most of the proposed solutions require complex and bulky spectroscopy systems, including xenon lamps, ultraviolet (UV) LASERs, monochromators, optical fibers, and high-quantum efficiency (QE) detectors. The consequence of using such components is the impossibility of their integration with the endoscopic equipment. Additionally, the literature refers to a few attempts [147,148] to potentiate the miniaturization by replacing a few of the components with photodiodes and/or light-emitting diodes (LEDs). However, this still requires a few pieces of macroscopic equipment.

**Figure 11:**
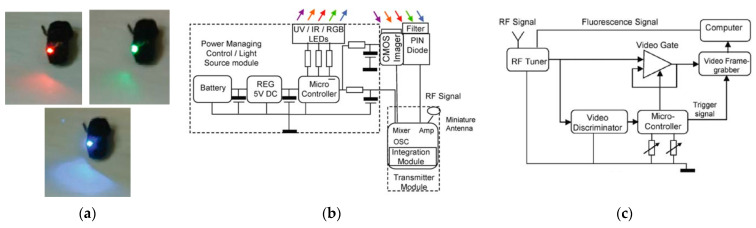
(**a**) Photograph of CPE with red, green, and blue LEDs. (**b**,**c**) Block diagrams of CPE module and receiving system, respectively [149].

One of the successful attempts to integrate spectroscopic techniques into endoscopic capsules can be tracked to the year of 2005 and credited to the research group of R. R. Alfano, which presents a fully working and tested prototype of a compact endoscopic capsule [149]. This medical device, named the Compact Photonics Explorer (CPE), is a further development of their work that was patented on 2001 [150] and is a complete endoscopic capsule solution composed of five light sources to cover the ultraviolet (UV), near-infra-red (NIR), and the entire visible spectrums (using three RGB LEDs). Figure 11 shows a photograph and block diagram of the current CPE and also the block diagram of the receiving station module. The spanning of these colors across these three bands increases the chance of early detection of lesions in the tissue, improving the chances of cancer treatment. Moreover, this endoscopic capsule is also composed of a CMOS image sensor, a PIN diode with an optical filter on top, a system to manage the energy supplied to the capsule, a radio-frequency transmission module, a microcontroller to perform core and control operations, and a small-sized battery. The effectiveness of the device was proven in reference [149] and further consolidated in 2011 through a US patent [151].

## 10. Narrow-Band Imaging

The idea of the NBI was conceived in 1999 by the Japanese National Cancer Center Hospital and Olympus Medical Systems (Tokyo, Japan) [132,152]. To confirm this idea, a study was conducted using a multispectral camera with a source of high-power light. In this study, Kazuhiro Gono volunteered himself to perform the first tests, and they revealed that the use of a narrow band at a wavelength of 415 nm could increase the contrast of the images of blood capillaries [153].

The NBI technology emerged from the need for detecting lesions that were not able to be observed in white light. Therefore, the technique is used to increase the endoscopic image contrast by capturing real-time images and using a system composed of cameras and optical filters.

The optical filters are positioned under the endoscope light to create a narrow-band wavelength in the blue (in the range of 400–430 nm, centered at 415 nm) and green (in the range of 530–550 nm, centered at 540 nm) [154].

In 2004, Machida et al. [155] described the first clinical utility of NBI for gastrointestinal endoscopy. The first release of the technique was in 2005 with a system developed by Olympus Medical Systems [153,156,157]. Since then, most of the countries that have endoscopy procedures have started applying NBI combined with the conventional techniques of endoscopy in clinical studies.

In 2003, Gono et al. conducted an experiment observing endoscopic images of the back mucosa of a human tongue and investigated the effect of NBI through preliminary clinical tests in upper and lower endoscopy [158]. The study showed that NBI could enhance the capillary and the crypt pattern on the mucosa, which are useful features for diagnosing early cancer [153,157,159].

These structures, such as blood vessels, have a high hemoglobin content, that is, hemoglobin index, which can be assessed by adjusting the color of the reflected light that penetrates the mucosa [160,161,162]. In this way, they appear darker, creating a higher contrast between the surrounding mucosa, which appears brighter when it reflects the light.

The blue light, at the wavelength of 415 nm, allows for the obtainment of a superficial mucosa image, in which it can be observed that the superficial capillaries network, while the green light, at the wavelength of 540 nm, allows for a deeper penetration in the mucosa, thus enabling the observation of subepithelial vessels. The photons, at a wavelength of 600 nm, that means, red light, are less scattered and penetrate more deeply. Although their longer wavelength is outside of the hemoglobin absorption band, the red photons reproduce a morphological image of the large vessels [157].

Moreover, the inclusion of a lens with a high magnification factor will further improve the image quality and detail, the global impact, and the importance of NBI [130].

In the context of NBI technology, there are two options for the implementation: it can use commercial LEDs and then adapt them with optical filters, or the LEDs can be fabricated with transmittance peaks at the wanted wavelength. The fabrication of optical filters offers a much cheaper alternative to the idea of fabricating LEDs with exact transmittance peaks.

Considering the scenario described before, it was decided to go for the option of fabricating the optical filters for adaptation into the commercial endoscopic capsules. To achieve this, different methods and processes have been tried until the final functional version of the filter was reached. The optical filters were first calculated, simulated and adjusted, and then fabricated.

### 10.1. Design and Fabrication of Optical Filters

As a first step, the materials from which the filters will be made should be chosen and specified. In this case, dielectric materials following the physical principle of Fabry–Perot were chosen, although there was also the possibility of working with metals. The filters consist of a double stack of layers, with a high-refractive-index material (H) and a low-refractive-index (L) material, alternately. The stacks of layers are called mirrors, and between both mirrors, there is a resonance cavity. This type of filter consists of a structure in which light is captured at certain wavelengths, and it operates as an optical transmission incorporating feedback: the light is repeatedly reflected between the two mirrors, without escaping. The transmitted light in the resonance cavity is the sum of all beams that are transmitted through the stack of layers due to a constructive interference, which depends on the wavelength of the incident light and the thickness of the resonance cavity.

The project of a Fabry–Perot filter requires selectivity and low absorption in the mirrors and in the resonance cavity. A slight change in the mirrors’ spacing can cause a significant change in the resulting wavelength. The optical filter is considered ideal if we assume that there is no loss on both mirrors and that they are perfectly parallel to each other [163,164]. Once the materials are chosen, the width of each layer of the mirror and of the resonance cavity should be calculated, based on the wavelength and refractive index we want for the filter. For the computational simulation, we used the software TFcalc^TM^ (3.5, Software Spectra, Inc., Vermillion, SD, USA). TFcalc^TM^ consists of a thin-film-design software, which enables the analysis and design of multilayer thin-film coatings, such as the calculation of transmittance, absorption, optical density, loss, color, and other features. These simulations took into account the spectrum of the optical sources. The optical filters were designed following a stack of seven layers, whose succession of refractive indexes follows the HLH-L-HLH structure. Therefore, the materials must be selected in order to provide high (H) and low (L) indexes of refraction in the visible range. The silicon dioxide (SiO_2_) and titanium dioxide (TiO_2_) were selected as H and L materials, because both can be obtained using physical processes, i.e., both can be deposited through sputtering, both are compatible in the sense that their mutual adhesion is high, and the silicon dioxide presents an index of refraction that is practically constant in the visible range [148]. The TiO_2_ and SiO_2_ present typical indexes of refraction of 1.45 and 2.65, respectively. In these filters, the three top layers correspond to the first mirror, and the three bottom layers correspond to the second mirror. Between the mirrors, there is a resonance cavity.

The optical filters were fabricated using a glass substrate B270 from Schott Advanced Optics, measuring 20 × 20 mm and with a thickness of 1 mm. The thin films of SiO_2_ and TiO_2_ were deposited by DC-magnetron sputtering and were previously characterized by ellipsometry in order to measure the refractive index and thin film thickness of both materials. The full fabrication details can be found in the work by Gounella et al. [165].

### 10.2. Optical Filter Characterization and Results

The optical filters were characterized via spectrometry, which allows for the evaluation of their optical performance. The characterization of transmittance was performed to know the optical response of the NBI filters to the fringes at 415 nm (blue region) and at 540 nm (green region).

As a first element of the setup, the illuminants (in this case, the blue and green LEDs) were placed before a collimator. The collimator played the role of narrowing the scattered beams from the LED and aligning them with the spectrometer. This step was necessary, since the aperture from the spectrometer was very narrow, which made the alignment difficult. For this experiment, a CCD compact spectrometer CCS200 from Thorlabs was used, with a wavelength range from 200 to 1000 nm.

**Figure 12:**
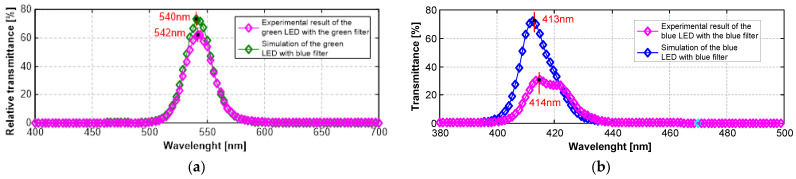
(**a**) Results of the green filter characterized via spectrometry. The green filter is centered at 542 nm with relative transmittance of 62%. (**b**) Results of the blue filter characterized via spectrometry. The blue filter is centered at 414 nm with relative transmittance of 33%.

Figure 12a shows the transmittance spectrum from the green optical filter, along with a picture of the physical filter fabricated. The green filter presented a maximum relative transmittance of 62% at 542 nm and a full width at half maximum (FWHM) of 29 nm. Figure 12b shows the experimental result of the blue filter with the blue LED as illuminant, along with a picture of the fabricated physical filter. The result showed a maximum relative transmittance of 33% at 414 nm and an FWHM of 17 nm. The peaks of transmittance were satisfactory once the goal was a blue filter centered at 415 nm and a green filter centered at 540 nm. The blue filter presented only 1 nm of deviation from the objective, despite the fact that the experimental transmittance was half of the simulated one. The green filter deviated 2 nm to the left from the objective, although also with higher transmittance at 540 nm (~60%).

### 10.3. Narrow-Band Imaging vs. CLE

The advantage of CLE lies in its ability to be used with any endoscopy system and visualize at a cellular level, thus lending itself to molecular imaging. On the other hand, it is also a limitation, since it cannot be used widely in community settings outside of academic centers [166].

### 10.4. Narrow-Band Imaging vs. FICE

Flexible spectral imaging color enhancement (FICE) and narrow-band imaging are forms of digital chromoendoscopy and enhance the endoscopic images without the need for a dye. Alis et al. found no statistically significant difference between them for in vivo histologic diagnosis of polyps in a study with 134 patients (72 male/62 female) [167].

### 10.5. Narrow-Band Imaging vs. BLI

Recently, a new optical imaging technology known as blue-light imaging (BLI; Fujifilm) was developed. The system uses either a laser or four LEDs with multilight technology to produce brighter images. It is the first nonfilter technology producing blue light in a narrow spectrum that is bright enough to identify subtle changes in surface and vessel patterns, which is of relevance in early Barrett’s neoplasia [168]. BLI magnification images with early gastric cancer are compared with those of NBI magnification images in deeply depressed areas, supported by a post-ESD (scopic submucosal dissection) specimen. NBI magnification cannot focus on the shallow area in a cancerous lesion very sharply when the deeply depressed area is observed in good focus. By contrast, BLI magnification can focus on both shallow and deeply depressed areas on the same image because of a great depth of field. This case shows a typically fine network pattern of early gastric cancer in the gastric body. BLI bright images show a fine network pattern in depressed cancer by exhibiting clearly irregular microvessels surrounding various sizes of white spots corresponding to histologically marginal crypt epithelium [169].

### 10.6. Narrow-Band Imaging, FICE, and BLI Images Enhanced by i-SCAN

**Figure 13:**
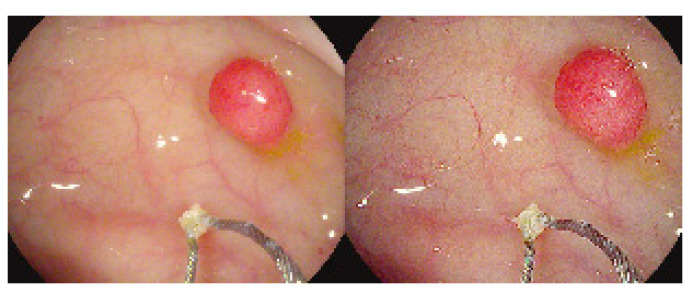
Comparison of images. (**Left**): raw image. (**Right**): i-SCAN engaged (Pentax whitepaper).

Developed by Pentax, i-SCAN is a dynamic, software-based image enhancement technology that provides the user an enhanced view of the texture of the mucosal surface and blood vessels. A study by Lee et al. presented results on i-SCAN vs. the more widely studied NBI for the prediction of diminutive colonic polyp pathology and found that both technologies had a statistically significant higher sensitivity and accuracy compared with BLI. In addition, no significant differences were evident between NBI and i-SCAN (sensitivity, 88.8% vs. 94.6%; specificity, 86.8% vs. 86.4%; accuracy, 87.8% vs. 90.7%, respectively; *p* < 0.05) [170]. Another study investigated i-SCAN’s role in evaluating duodenal villous patterns [171]. Figure 13 compares the quality of an image enhanced by the i-SCAN technology (on right) with a raw image (on left).

## 11. Conclusions

The aim of this research paper is to present a detailed review of the current status of endoscopic techniques and the recent technological advancements that can be employed in conjunction with these techniques in order to improve a timely diagnosis of gastrointestinal disorders. Furthermore, this study highlights the design and fabrication of two optical filters, which have been created with the specific purpose of enhancing the detection of hemoglobin in the gastrointestinal tract. These filters have been centered at wavelengths of 414 nm and 542 nm, which coincide with the absorption peaks of hemoglobin. In order to create these optical filters, narrow-band imaging (NBI) technology has been utilized. The ultimate objective of this effort is to integrate these filters into the endoscopic capsules for practical applications. Despite the challenges associated with the fabrication of these optical filters, the results of the experimental studies have been satisfactory when compared with the simulations. The complexity and difficulty of the manufacturing process have been taken into account while evaluating the outcomes of the study. The future advances in diagnostic endoscopy now lie in combinations of these new optical techniques along with improvements from the field of artificial intelligence to minimize human error and maximize its efficiency, finally enabling the automatic screening diagnosis predicted by the companies in the past.

## Figures and Tables

**Table 1:** Commercial capsules.

Capsule	Company	Indication	Imaging System	Size (Diam. × Length)	References
PillCam SB	Given Imaging Inc.	For pediatric patients; diagnosis of small bowel pathologies	1 video camera; 2 fps	11.4 mm × 26.4 mm	[65,91]
PillCam SB2	Given Imaging Inc.	For diagnosis of small bowel pathologies	1 video camera with fixed high frame rate; 2 or 4 fps	11.4 mm × 26.3 mm	[92]
PillCam SB3	Given Imaging Inc.	For diagnosis of small bowel pathologies	1 video camera; enhanced imaging capabilities and AFR; 2 fps or 2–6 fps	11.4 mm × 26.2 mm	[92]
PillCam ESO 2	Given Imaging Inc.	For investigation of esophageal disorders	2 video cameras; 19 fps	11.4 mm × 26.4 mm	[92,93,94,95]
PillCam ESO 3	Given Imaging Inc.	For investigation of esophageal disorders	2 video cameras; 35 fps	11.6 mm × 31.5 mm	[92,93,94,95]
PillCam COLON	Given Imaging Inc.	For investigation of intestinal disorders	2 video cameras and AFR; 4 fps	11.4 mm × 31 mm	[92]
PillCam COLON 2	Given Imaging Inc.	For investigation of intestinal disorders	2 video cameras and AFR; 4–35 fps	11.6 mm × 31.5 mm	[92]
Patency System	Given Imaging Inc.	For detecting obstructions or strictures in the GI tract	None (radiofrequency identification (RFID) detected by an RFID scanner)	11.4 mm × 26.4 mm	[97]
OMOM	Jinshan Science and Technology Company	For investigation of OGIB, abdominal pain or diarrhea, inflammatory bowel disease, and tumors	1 video camera; 2 fps	13 mm × 27.9 mm	[99]
EndoCapsule	Olympus Medical Systems	Detection of GI bleeding	1 video camera and high-resolution image chip	11 mm × 26 mm	[28,100,101,102]
MiRoCam	Intromedic	For investigation of stomach, small bowel, and colon	1 video camera; 2 fps	10.8 mm × 24 mm	[102,104]

## References

[1] Moltzer E., Noordman B.J., Renken N.S., Roos D. (2019). Determination of Tumor Location in Rectosigmoid Carcinomas: Difficulties in Preoperative Diagnostics. Gastrointest. Disord..

[2] Ortega S., Fabelo H., Iakovidis D.K., Koulaouzidis A., Callico G.M. (2019). Use of Hyperspectral/Multispectral Imaging in Gastroenterology. Shedding Some–Different–Light into the Dark. J. Clin. Med..

[3] Beeley J., Melino G., Al-Rawahani M., Turcanu M., Stewart F., Cochran S., Cumming D. (2018). Imaging Fluorophore-Labelled Intestinal Tissue via Fluorescence Endoscope Capsule. Proceedings.

[4] Nakamura M., Yamamura T., Maeda K., Sawada T., Mizutani Y., Ishikawa T., Furukawa K., Ohno E., Kawashima H., Miyahara R. (2018). Validity of Capsule Endoscopy in Monitoring Therapeutic Interventions in Patients with Crohn’s Disease. J. Clin. Med..

[5] Sahoo G.R., Singh P., Pandey K., Kala C., Pradhan A. (2018). Improving Diagnosis of Cervical Pre-Cancer: Combination of PCA and SVM Applied on Fluorescence Lifetime Images. Photonics.

[6] Askoura M.L., Vaudelle F., L’Huillier J.P. (2016). Experimental Study of Light Propagation in Apple Tissues Using a Multispectral Imaging System. Photonics.

[7] Rohrbach D.J., Salem H., Aksahin M., Sunar U. (2016). Photodynamic Therapy-Induced Microvascular Changes in a Nonmelanoma Skin Cancer Model Assessed by Photoacoustic Microscopy and Diffuse Correlation Spectroscopy. Photonics.

[8] Peretti R., Braud F., Peytavit E., Dubois E., Lampin J.F. (2018). Broadband Terahertz Light-Matter Interaction Enhancement for Precise Spectroscopy of Thin Films and Micro-Samples. Photonics.

[9] Tansu N. (2014). Photonics-Advances in Fundamental Sciences and Engineering Technologies of Light. Photonics.

[10] Chiu P.W.Y. (2013). Second Look Endoscopy in Acute Non-Variceal Upper Gastrointestinal Bleeding. Best Pract. Res. Clin. Gastroenterol..

[11] Kahi C.J., Myers L.J., Slaven J.E., Haggstrom D., Pohl H., Robertson D.J., Imperiale T.F. (2014). Lower Endoscopy Reduces Colorectal Cancer Incidence in Older Individuals. Gastroenterology.

[12] Pan G., Wang L. (2012). Swallowable Wireless Capsule Endoscopy: Progress and Technical Challenges. Gastroenterol. Res. Pract..

[13] Karargyris A., Bourbakis N. (2010). Wireless Capsule Endoscopy and Endoscopy Imaging. IEEE Eng. Med. Biol..

[14] Iddan G., Meron G., Glukhovsky A., Swain P. (2000). Wireless Capsule Endoscopy. Nature.

[15] Li F., Leighton J.A., Sharma V.K. (2007). Capsule Endoscopy in the Evaluation of Obscure Gastrointestinal Bleeding: A Comprehensive Review. Gastroenterol. Hepatol..

[16] Schnoll-Sussman F., Kulkarni K. (2008). Risks of Capsule Endoscopy. Tech. Gastrointest. Endosc..

[17] Rondonotti E., Herrerias J.M., Pennazio M., Caunedo A., Mascarenhas-Saraiva M., De Franchis R. (2005). Complications, Limitations, and Failures of Capsule Endoscopy: A Review of 733 Cases. Gastrointest. Endosc..

[18] Spada C., Shah S.K., Riccioni M.E., Spera G., Marchese M., Iacopini F., Familiari P., Costamagna G. (2007). Video Capsule Endoscopy in Patients With Known or Suspected Small Bowel Stricture Previously Tested With the Dissolving Patency Capsule. J. Clin. Gastroenterol..

[19] Boivin M.L., Lochs H., Voderholzer W.A. (2005). Does Passage of a Patency Capsule Indicate Small−Bowel Patency? A Prospective Clinical Trial?. Endoscopy.

[20] Signorelli C., Rondonotti E., Villa F., Abbiati C., Beccari G., Avesani E.C., Vecchi M., de Franchis R. (2006). Use of the Given^®^ Patency System for the Screening of Patients at High Risk for Capsule Retention. Dig. Liver Dis..

[21] Holden J.P., Dureja P., Pfau P.R., Schwartz D.C., Reichelderfer M., Judd R.H., Danko I., Iyer L.V., Gopal D.V. (2007). Endoscopic Placement of the Small-Bowel Video Capsule by Using a Capsule Endoscope Delivery Device. Gastrointest. Endosc..

[22] Selby W., Sydney F. (2005). Complete Small-Bowel Transit in Patients Undergoing Capsule Endoscopy: Determining Factors and Improvement with Metoclopramide. Gastrointest. Endosc..

[23] Fireman Z., Paz D., Elsevier K., Kopelman Y. (2005). Capsule Endoscopy: Improving Transit Time and Image View. World J. Gastroenterol..

[24] Moy L., Levine J. (2007). Wireless Capsule Endoscopy in the Pediatric Age Group: Experience and Complications. J. Pediatr. Gastroenterol. Nutr..

[25] Ben-Soussan E., Savoye G., Antonietti M., Ramirez S., Ducrotté P., Lerebours E. (2005). Is a 2-Liter PEG Preparation Useful Before Capsule Endoscopy?. J. Clin. Gastroenterol..

[26] Dai N., Gubler C., Hengstler P., Meyenberger C., Bauerfeind P., Gallen S. (2005). Improved Capsule Endoscopy after Bowel Preparation. Gastrointest. Endosc..

[27] Shiotani A., Opekun A.R., Graham D.Y. (2007). Visualization of the Small Intestine Using Capsule Endoscopy in Healthy Subjects. Dig. Dis. Sci..

[28] Pennazio M. (2006). Capsule Endoscopy: Where Are We after 6 Years of Clinical Use?. Dig. Liver Dis..

[29] Jain R.K., Jain S., Pascu O., Seicean A. (2011). Capsule Endoscopy: A Comprehensive Review. New Techniques in Gastrointestinal Endoscopy.

[30] Leighton J.A., Sharma V.K., Srivathsan K., Heigh R.I., McWane T.L., Post J.K., Robinson S.R., Bazzell J.L., Fleischer D.E. (2004). Safety of Capsule Endoscopy in Patients with Pacemakers. Gastrointest. Endosc..

[31] Leighton J.A., Srivathsan K., Carey E.J., Sharma V.K., Heigh R.I., Post J.K., Erickson P.J., Robinson S.R., Bazzell J.L., Fleischer D.E. (2005). Safety of Wireless Capsule Endoscopy in Patients with Implantable Cardiac Defibrillators. Off. J. Am. Coll. Gastroenterol. |ACG.

[32] Swain P. (2008). The future of wireless capsule endoscopy. World J. Gastroenterol..

[33] Qureshi W.A. (2004). Current and future applications of the capsule camera. Nat. Rev. Drug Discov..

[34] Carta R., Pateromichelakis N., Thoné J., Sfakiotakis M., Tsakiris D.P., Puers R. A wireless powering system for a vibratory-actuated endoscopic capsule. Proceedings of the Eurosensors XXII.

[35] Sfakiotakis M., Tsakiris D.P. Pedundulatory robotic locomotion: Centipede and polychaete modes in unstructured substrates. Proceedings of the IEEE International Conference on Robotics and Biomimetics (ROBIO’08).

[36] Valdastri P., Webster R.J., Quaglia C., Quirini M., Menciassi A., Dario P. (2009). A new mechanism for meso-scale legged locomotion in compliant tubular environments. IEEE Trans. Robot..

[37] Carta R., Tortora G., Thoné J., Lenaerts B., Valdastri P., Menciassi A., Dario P., Puers R. (2009). Wireless powering for a self-propelled and steerable endoscopic capsule for stomach inspection. Biosens. Bioelectron..

[38] Roth Net Standard Retrievers, US Endoscopy. www.usendoscopy.com.

[39] Zabulis X., Sfakiotakis M., Tsakiris D.I. Effects of vibratory actuation on endoscopic capsule vision. Proceedings of the 30th Annual International Conference of the IEEE Engineering in Medicine and Biology Society.

[40] Radley B., Elson J.N., Gervasoni S., Chiu P.W.Y., Hang L., Zemmar A. (2022). Magnetically Actuated Medical Robots: An in vivo Perspective. Proc. IEEE.

[41] Carpi F., Galbiati S., Carpi A. (2007). Controlled Navigation of Endoscopic Capsules: Concept and Preliminary Experimental Investigations. IEEE Trans. Biomed. Eng..

[42] Silva M.F., Ribeiro J.F., Goncalves L.M., Carmo J.P., Silva C.A., Correia J.H. (2011). Magnetic control platform for wireless endoscopic capsules. Procedia Eng..

[43] Keller H., Juloski A., Kawano H., Bechtold M., Kimura A., Takizaw H. Method for navigation and control of a magnetically guided capsule endoscope in the human stomach. Proceedings of the 4th IEEE RAS & EMBS International Conference on Biomedical Robotics and Biomechatronics (BioRob).

[44] Tai C., Chen M., Luo C. (2007). Study of a magnetic levitation technique for use in a wireless capsule endoscope. J. Med. Biol. Eng..

[45] Shaheen N.J., Richter J.E. (2009). Barrett’s Oesophagus. Lancet.

[46] Spechler S.J. (2002). Barrett’s Esophagus. New Engl. J. Med..

[47] Shaheen N.J., Crosby M.A., Bozymski E.M., Sandler R.S. (2000). Is There Publication Bias in the Reporting of Cancer Risk in Barrett’s Esophagus?. Gastroenterology.

[48] Blot W.J., Devesa S.S., Kneller R.W., Fraumeni J.F. (1991). Rising Incidence of Adenocarcinoma of the Esophagus and NGastric Cardia. JAMA.

[49] Devesa S.S., Blot W.J., Fraumeni J.F. (1998). Changing Patterns in the Incidence of Esophageal and Gastric Carcinoma in the United States. Cancer.

[50] Galmiche J.P., Coron E., Sacher-Huvelin S. (2008). Recent Developments in Capsule Endoscopy. Gut.

[51] Gerson L., Lin O.S. (2007). Cost-Benefit Analysis of Capsule Endoscopy Compared With Standard Upper Endoscopy for the Detection of Barrett’s Esophagus. Clin. Gastroenterol. Hepatol..

[52] Alsayid M., Melson J. (2020). Will magnet-assisted capsule endoscopy become a viable screening tool for Barrett’s esophagus and esophageal varices?. Clin. Endosc..

[53] Pena L.R., Cox T., Koch A.G., Bosch A. (2008). Study Comparing Oesophageal Capsule Endoscopy versus EGD in the Detection of Varices. Dig. Liver Dis..

[54] McCarty T.R., Afinogenova Y., Njei B. (2017). Use of Wireless Capsule Endoscopy for the Diagnosis and Grading of Esophageal Varices in Patients With Portal Hypertension: A Systematic Review and Meta-Analysis. J. Clin. Gastroenterol..

[55] Eisen R., Zaman A., Schwartz J., Faigel D., Rondonotti E., Villa F., Weizman E., Yassin K., deFranchis R. (2006). The Accuracy of PillCam ESO Capsule Endoscopy Versus Conventional Upper Endoscopy for the Diagnosis of Esophageal Varices: A Prospective Three-Center Pilot Study. Endoscopy.

[56] Lu Y., Gao R., Liao Z., Hu L.H., Li Z.S. (2009). Meta-Analysis of Capsule Endoscopy in Patients Diagnosed or Suspected with Esophageal Varices. World J. Gastroenterol.

[57] Beg S., Card T., Warburton S., Rahman I., Wilkes E., White J., Ragunath K. (2020). Diagnosis of Barrett’s esophagus and esophageal varices using a magnetically assisted capsule endoscopy system. Gastrointest. Endosc..

[58] Jensen D.M., Singh B., Chavalitdhamrong D., Kovacs T.O., Carrico M., Han S.-H.B., Durazo F.A., Saab S. (2008). Is Capsule Endoscopy Accurate Enough to Screen Cirrhotics for High Risk Varices & Other Lesions? A Blinded Comparison of EGD & PillCam ESO. Gastrointest. Endosc..

[59] Smith R.A., Cokkinides V., von Eschenbach A.C., Levin B., Cohen C., Runowicz C.D., Sener S., Saslow D., Eyre H.J. (2002). American Cancer Society Guidelines for the Early Detection of Cancer. CA Cancer J. Clin..

[60] Cobrin G.M., Pittman R.H., Lewis B.S. (2006). Increased Diagnostic Yield of Small Bowel Tumors with Capsule Endoscopy. Cancer.

[61] Delvaux M., Gay G. (2008). Capsule Endoscopy: Technique and Indications. Best Pract. Res. Clin. Gastroenterol..

[62] Alquist D., Fennerty B., Fleischer D., McDonnell W.M., McGill D.B., Waring P., Wilcox C.M., Winawer S. (2000). American Gastroenterological Association Medical Position Statement: Evaluation and Management of Occult and Obscure Gastrointestinal Bleeding. Gastroenterology.

[63] Rockey D.C. (2010). Occult and Obscure Gastrointestinal Bleeding: Causes and Clinical Management. Nat. Rev. Gastroenterol. Hepatol..

[64] Pennazio M., Santucci R., Rondonotti E., Abbiati C., Beccari G., Rossini F.P., De Franchis R. (2004). Outcome of Patients with Obscure Gastrointestinal Bleeding after Capsule Endoscopy: Report of 100 Consecutive Cases. Gastroenterology.

[65] Triester S.L., Leighton J.A., Leontiadis G.I., Gurudu S.R., Fleischer D.E., Hara A.K., Heigh R.I., Shiff A.D., Sharma V.K. (2006). A Meta-Analysis of the Yield of Capsule Endoscopy Compared to Other Diagnostic Modalities in Patients with Non-Stricturing Small Bowel Crohn’s Disease. Off. J. Am. Coll. Gastroenterol. |ACG.

[66] Yamamoto H., Sekine Y., Sato Y., Higashizawa T., Miyata T., Iino S., Ido K., Sugano K. (2001). Total Enteroscopy with a Nonsurgical Steerable Double-Balloon Method. Gastrointest. Endosc..

[67] Rondonotti E., Koulaouzidis A., Silvia P., Franco R., Pennazio M. (2015). Obscure Gastrointestinal Bleeding and Iron-Deficiency Anemia-Where Does Capsule Endoscopy Fit?. Tech. Gastrointest. Endosc..

[68] Lennard-Jones J.E. (1989). Classification of Inflammatory Bowel Disease. Scand J. Gastroenterol..

[69] Fornaro R., Frascio M., Denegri A., Stabilini C., Imperatore M., Mandolfino F., Fabrizio L., Gianetta E. (2009). Malattia Di Crohn e Cancro. Ann. Ital. Chir..

[70] Baumgart D.C., Sandborn W.J. (2012). Crohn’s Disease. Lancet.

[71] Schulmann K., Hollerbach S., Schmiegel W. (2003). Diagnosing Small Bowel Crohn’s Disease with Wireless Capsule Endoscopy. Gut.

[72] Albert J.G., Martiny F., Krummenerl A., Stock K., Leßke J., Göbel C.M., Lotterer E., Nietsch H.H., Behrmann C., Fleig W.E. (2005). Diagnosis of Small Bowel Crohn’s Disease: A Prospective Comparison of Capsule Endoscopy with Magnetic Resonance Imaging and Fluoroscopic Enteroclysis. Gut.

[73] Odeyinka O., Alhashimi R., Thoota S. (2022). The Role of Capsule Endoscopy in Crohn’s Disease: A Review. Cureus.

[74] Leighton J.A., Helper D.J., Gralnek I.M., Dotan I., Fernandez-Urien I., Lahat A., Malik P., Mullin G.E., Rosa B. (2017). Comparing Diagnostic Yield of a Novel Pan-Enteric Video Capsule Endoscope with Ileocolonoscopy in Patients with Active Crohn’s Disease: A Feasibility Study. Gastrointest. Endosc..

[75] Fry L., Carey E., Shiff A., Heigh R., Sharma V., Post J., Hentz J., Fleischer D., Leighton J. (2006). The Yield of Capsule Endoscopy in Patients with Abdominal Pain or Diarrhea. Endoscopy.

[76] Petroniene R., Dubcenco E., Baker J.P., Ottaway C.A., Tang S.-J., Zanati S.A., Streutker C.J., Gardiner G.W., Warren R.E., Jeejeebhoy K.N. (2005). Given^®^ Capsule Endoscopy in Celiac Disease: Evaluation of Diagnostic Accuracy and Interobserver Agreement. Off. J. Am. Coll. Gastroenterol.|ACG.

[77] Green P.H.R., Rubin M. (2005). Capsule Endoscopy in Celiac Disease. Gastrointest. Endosc..

[78] Lewis S.K., Semrad C.E. (2019). Capsule Endoscopy and Enteroscopy in Celiac Disease. Gastroenterol. Clin. North Am..

[79] Luján-Sanchis M., Pérez-Cuadrado-Robles E., García-Lledó J., Fernández J.F.J., Elli L., Jiménez-García V.A., Egea-Valenzuela J., Valle-Muñoz J., Carretero-Ribón C., Fernández-Urién-Sainz I. (2017). Role of Capsule Endoscopy in Suspected Celiac Disease: A European Multi-Centre Study. World J. Gastroenterol..

[80] Cellier P.H.R., Collin P., Murray J. (2005). ICCE Consensus for Celiac Disease. Endoscopy.

[81] Soares L., Vilas Boas G., Pinho C.J.L. (2004). Wireless Capsule Endoscopy for Evaluation of Phenotypic Expression of Small-Bowel Polyps in Patients with Peutz-Jeghers Syndrome and in Symptomatic First-Degree Relatives. Endoscopy.

[82] Swain C.P., Gong F., Mills T.N. (1997). Wireless Transmission of a Colour Television Moving Image from the Stomach Using a Miniature CCD Camera, Light Source and Microwave Transmitter. Gastrointest. Endosc..

[83] Appleyard M., Glukhovsky A., Swain P. (2001). Wireless-Capsule Diagnostic Endoscopy for Recurrent Small-Bowel Bleeding. N. Engl. J. Med..

[84] Kornbluth A., Legnani P., Lewis B.S. (2004). Video Capsule Endoscopy in Inflammatory Bowel Disease: Past, Present, and Future. Inflamm. Bowel. Dis..

[85] Cave D., Legnani P., De Franchis R., Lewis B.S. (2005). ICCE Consensus for Capsule Retention. Endoscopy.

[86] Lewis B.S., Rey J.F., Seidman E.G. (2005). Capsule Endoscopy 2005: Results of the 2005 International Consensus Conference–Introduction. Endoscopy.

[87] De Franchis A., Barkin J., Cave D., Filoche B.R.A. (2005). ICCE Consensus for Bowel Preparation and Prokinetics. Endoscopy.

[88] Pennazio G., Goldfarb N.M.E. (2005). ICCE Consensus for Obscure Gastrointestinal Bleeding. Endoscopy.

[89] Kornbluth J.F., Leighton J.A., Loftus E.A.C. (2005). ICCE Consensus for Inflammatory Bowel Disease. Endoscopy.

[90] Sharma R., Sharma P., Faigel D.V.K.E. (2005). ICCE Consensus for Esophageal Capsule Endoscopy. Endoscopy.

[91] Gurudu S.R., Vargas H.E., Leighton J.A. (2008). New Frontiers in Small-Bowel Imaging: The Expanding Technology of Capsule Endoscopy and Its Impact in Clinical Gastroenterology. Rev. Gastroenterol. Disord..

[92] PillCamTM SB 3 Capsule Endoscopy System|Medtronic. https://www.medtronic.com/covidien/en-us/products/capsule-endoscopy/pillcam-sb3-system.html.

[93] Galmiche J.P., Sacher-Huvelin S., Coron E., Cholet F., Soussan E.B., Sébille V., Filoche B., D’Abrigeon G., Antonietti M., Robaszkiewicz M. (2008). Screening for Esophagitis and Barrett’s Esophagus with Wireless Esophageal Capsule Endoscopy: A Multicenter Prospective Trial in Patients with Reflux Symptoms. Am. J. Gastroenterol..

[94] Eliakim R., Yassin K., Shlomi I., Suissa A., Eisen G.M. (2004). A Novel Diagnostic Tool for Detecting Oesophageal Pathology: The PillCam Oesophageal Video Capsule. Aliment. Pharmacol. Ther..

[95] Eliakim R., Sharma V.K., Yassin K., Adler S.N., Jacob H., Cave D.R., Sachdev R., Mitty R.D., Hartmann D., Schilling D. (2005). A Prospective Study of the Diagnostic Accuracy of PillCam ESO Esophageal Capsule Endoscopy versus Conventional Upper Endoscopy in Patients with Chronic Gastroesophageal Reflux Diseases. J. Clin. Gastroenterol..

[96] Römmele C., Brueckner J., Messmann H., Gölder S.K. (2016). Clinical Experience with the PillCam Patency Capsule Prior to Video Capsule Endoscopy: A Real-World Experience. Gastroenterol. Res. Pract..

[97] Spada C., Spera G., Riccioni M., Biancone L., Petruzziello L., Tringali A., Familiari P., Marchese M., Onder G., Mutignani M. (2005). A Novel Diagnostic Tool for Detecting Functional Patency of the Small Bowel: The given Patency Capsule. Endoscopy.

[98] Caunedo-Álvarez Á., Romero-Vazquez J., Herrerias-Gutierrez J.M. (2008). Patency© and Agile© Capsules. World J. Gastroenterol..

[99] Li C.Y., Zhang B.L., Chen C.X., Li Y.M. (2008). OMOM Capsule Endoscopy in Diagnosis of Small Bowel Disease. J. Zhejiang Univ. Sci. B.

[100] Moglia A., Menciassi A., Schurr M.O., Dario P. (2007). Wireless Capsule Endoscopy: From Diagnostic Devices to Multipurpose Robotic Systems. Biomed. Microdevices.

[101] Bang S., Park J.Y., Jeong S., Kim Y.H., Shim H.B., Kim T.S., Lee D.H., Song S.Y. (2009). First Clinical Trial of the “MiRo” Capsule Endoscope by Using a Novel Transmission Technology: Electric-Field Propagation. Gastrointest. Endosc..

[102] Capsule Endoscopy|ENDOCAPSULE 10 System|Olympus Medical Systems. https://www.olympus.co.uk/medical/en/Products-and-solutions/Products/Product/ENDOCAPSULE-10-System.html.

[103] Hartmann D., Eickhoff A., Damian U., Riemann J.F. (2007). Diagnosis of Small-Bowel Pathology Using Paired Capsule Endoscopy with Two Different Devices: A Randomized Study. Endoscopy.

[104] Mussetto A., Fuccio L., Dari S., Gasperoni S., Cantoni F., Brancaccio M.L., Triossi O., Casetti T. (2013). MiroCam Capsule for Obscure Gastrointestinal Bleeding: A Prospective, Single Centre Experience. Dig. Liver Dis..

[105] Esaki M., Matsumoto T., Matsui T., Matsumoto T., Aoyagi K. (2014). Capsule Endoscopy. Endoscopy in the Diagnosis of Small Intestine Diseases.

[106] Uchiyama A., Takizawa H., Yokoi T., Mizuno H. (2003). Encapsulated Endoscope System in Which Endoscope Moves in Lumen by Itself and Rotation of Image of Region to Be Observed Is Ceased. U.S. Patent.

[107] Hu C., Gao M., Chen Z., Zhang H., Liu S. Magnetic Analysis and Simulations of a Self-Propelled Capsule Endoscope. Proceedings of the 2010 11th International Conference on Thermal, Mechanical and Multi-Physics Simulation, and Experiments in Microelectronics and Microsystems, EuroSimE 2010.

[108] Matsui T., Murata S., Honda T. (2018). Fabrication of Magnetically Driven Biopsy Mechanism Applicable to Capsule-Type Medical Device. J. Robot. Mechatron..

[109] Carpi F., Galbiati S., Carpi A. (2006). Magnetic shells for gastrointestinal endoscopic capsules as a means to control their motion. Biomed Pharmacother..

[110] Casanovas O.A. (2012). Enabling Active Locomotion and Advanced Features in Capsule Endoscopy.

[111] Daniell M.D., Hill J.S. (1991). A History of Photodynamic Therapy. Aust. New Zealand J. Surg..

[112] Ackroyd R., Kelty C., Brown N., Reed M. (2007). The History of Photodetection and Photodynamic Therapy. Photochem Photobiol.

[113] Dolmans D.E.J.G.J., Fukumura D., Jain R.K. (2003). Photodynamic Therapy for Cancer. Nat. Rev. Cancer.

[114] Dhaneshwar S., Patil K., Bulbule M., Kinjawadekar V., Joshi D., Joshi V. (2014). Photodynamic Therapy for Cancer. Int. J. Pharm. Sci Rev. Res..

[115] Weishaupt K.R., Gomer C.J., Dougherty T.J. (1976). Identification of Singlet Oxygen as the Cytotoxic Agent in Photo-Inactivation of a Murine Tumor. Cancer Res..

[116] Triesscheijn M., Baas P., Schellens J.H.M., Stewart F.A. (2006). Photodynamic Therapy in Oncology. Oncologist.

[117] McCaughan J.S., Hicks W., Laufman L., May E., Roach R. (1984). Palliation of Esophageal Malignancy with Photoradiation Therapy. Cancer.

[118] Schweitzer V.G., Bologna S., Batra S.K. (1993). Head and Neck and Plastic Surgery A Targeted Problem and Its Solution. Laryngoscope.

[119] Qumseya B.J., David W., Wolfsen H.C. (2013). Photodynamic Therapy for Barrett’s Esophagus and Esophageal Carcinoma. Clin. Endosc..

[120] Moghissi K., Dixon K., Thorpe J.A.C., Stringer M., Moore P.J. (2000). The Role of Photodynamic Therapy (PDT) in Inoperable Oesophageal Cancer. Eur. J. Cardio-Thorac. Surg..

[121] Sibille A., Lambert R., Souquet J.C., Sabben G., Descos F. (1995). Long-Term Survival after Photodynamic Therapy for Esophageal Cancer. Gastroenterology.

[122] Grosjean P., Savary J.F., Mizeret J., Wagnieres G., Woodtli A., Theumann J.F., Fontolliet C., Van Den Bergh H., Monnier P. (1996). Photodynamic Therapy for Cancer of the Upper Aerodigestive Tract Using Tetra(m-Hydroxyphenyl)Chlorin. J. Clin. Laser Med. Surg..

[123] Brown L.M., Devesa S.S. (2002). Epidemiologic Trends in Esophageal and Gastric Cancer in the United States. Surg. Oncol. Clin. N Am..

[124] Overholt B.F., Panjehpour M. (1996). Photodynamic Therapy in Barrett’s Esophagus. J. Clin. Laser Med. Surg..

[125] Overholt B.F., Panjehpour M., Haydek J.M. (1999). Photodynamic Therapy for Barrett’s Esophagus: Follow-up in 100 Patients. Gastrointest. Endosc..

[126] Costa C.G., Gomes J.M., Wolffenbuttel R.F., Correia J.H. (2016). Optical Microsystem Design and Fabrication for Medical Image Magnification. Microsyst. Technol..

[127] Gounella R.H., Leite I.S., Inada N.M., Do Carmo J.P.P. (2020). Wireless Portable Evaluation Platform for Photodynamic Therapy: In Vitro Assays on Human Gastric Adenocarcinoma Cells. IEEE Sens. J..

[128] Teubner D., Kiesslich R., Matsumoto T., Rey J.W., Hoffman A. (2014). Beyond Standard Image-Enhanced Endoscopy Confocal Endomicroscopy. Gastrointest. Endosc. Clin. N Am..

[129] Clark C., Turner J. (2015). Diagnostic Modalities for Inflammatory Bowel Disease. Serologic Markers and Endoscopy. Surg. Clin. North Am..

[130] Ribeiro J.F., Costa A.C., Gomes J.M., Costa C.G., Goncalves S.B., Wolffenbuttel R.F., Correia J.H. (2017). PDMS Microlenses for Optical Biopsy Microsystems. IEEE Trans. Ind. Electron..

[131] Tabatabaei N., Kang D., Wu T., Kim M., Carruth R.W., Leung J., Sauk J.S., Shreffler W., Yuan Q., Katz A. (2014). Tethered Confocal Endomicroscopy Capsule for Diagnosis and Monitoring of Eosinophilic Esophagitis. Biomed. Opt. Express.

[132] Hou R., Le T., Murgu S.D., Chen Z., Brenner M. (2011). Recent Advances in Optical Coherence Tomography for the Diagnoses of Lung Disorders. Expert Rev. Respir. Med..

[133] Bouma B.E., Tearney G.J. (2002). Handbook of Optical Coherence Tomography.

[134] Pawley J.B., Masters B.R. (2008). Handbook of Biological Confocal Microscopy, Third Edition. J. Biomed. Opt..

[135] Gora M.J., Quénéhervé L., Carruth R.W., Lu W., Rosenberg M., Sauk J.S., Fasano A., Lauwers G.Y., Nishioka N.S., Tearney G.J. (2018). Tethered Capsule Endomicroscopy for Microscopic Imaging of the Esophagus, Stomach, and Duodenum without Sedation in Humans (with Video). Gastrointest. Endosc..

[136] Maciel M.J., Costa C.G., Silva M.F., Peixoto A.C., Wolffenbuttel R.F., Correia J.H. (2016). A Wafer-Level Miniaturized Michelson Interferometer on Glass Substrate for Optical Coherence Tomography Applications. Sens. Actuators A Phys..

[137] Maciel M.J., Rosa C.C., Wolffenbuttel R.F., Correia J.H. (2018). Optical Coherence Tomography within a Single Microsystem. J. Phys. D Appl. Phys..

[138] Pimenta S., Castanheira E.M.S., Minas G. (2015). Optical Microsystem for Analysis of Diffuse Reflectance and Fluorescence Signals Applied to Early Gastrointestinal Cancer Detection. Sensors.

[139] Yu C.-C., Lau C., O’Donoghue G., Mirkovic J., McGee S., Galindo L., Elackattu A., Stier E., Grillone G., Badizadegan K. (2008). Quantitative Spectroscopic Imaging for Non-Invasive Early Cancer Detection. Opt. Express.

[140] Georgakoudi I. (2006). The Color of Cancer. J. Lumin..

[141] Brown J.Q., Vishwanath K., Palmer G.M., Ramanujam N. (2009). Advances in Quantitative UV-Visible Spectroscopy for Clinical and Pre-Clinical Application in Cancer. Curr. Opin. Biotechnol..

[142] Pimenta S., Carmo J.P., Correia R.G., Minas G., Castanheira E.M.S. Characterization of Silicon Photodiodes for Diffuse Reflectance Signal Extraction. Proceedings of the Proceedings-2015 IEEE 4th Portuguese Meeting on Bioengineering, ENBENG 2015.

[143] Ell C. (2003). Improving Endoscopic Resolution and Sampling: Fluorescence Techniques. Gut.

[144] Georgakoudi I., Jacobson B.C., Van Dam J., Backman V., Wallace M.B., Müller M.G., Zhang Q., Badizadegan K., Sun D., Thomas G.A. (2001). Fluorescence, Reflectance, and Light-Scattering Spectroscopy for Evaluating Dysplasia in Patients with Barrett’s Esophagus. Gastroenterology.

[145] Mayinger B., Jordan M., Horner P., Gerlach C., Muehldorfer S., Bittorf B.R., Matzel K.E., Hohenberger W., Hahn E.G., Guenther K. (2003). Endoscopic Light-Induced Autofluorescence Spectroscopy for the Diagnosis of Colorectal Cancer and Adenoma. J. Photochem. Photobiol. B.

[146] Liu N.R., Chen G.N., Wu S.S., Chen R. (2014). Distinguishing Human Normal or Cancerous Esophagus Tissue Ex Vivo Using Multiphoton Microscopy. J. Opt. (UK).

[147] Lo J.Y., Yu B., Fu H.L., Bender J.E., Palmer G.M., Kuech T.F., Ramanujam N. (2009). A Strategy for Quantitative Spectral Imaging of Tissue Absorption and Scattering Using Light Emitting Diodes and Photodiodes. Opt Express.

[148] Yu B., Lo J.Y., Kuech T.F., Palmer G.M., Bender J.E., Ramanujam N. (2008). Cost-Effective Diffuse Reflectance Spectroscopy Device for Quantifying Tissue Absorption and Scattering in Vivo. J. Biomed. Opt..

[149] Wang L., Zhang G., Luo J.C., Zeng F., Wang Q.Z., Alfano S.A., Katz A., Zevallos M., Alfano R.R. (2005). Wireless Spectroscopic Compact Photonic Explorer for Diagnostic Optical Imaging. Biomed Microdevices.

[150] Alfano R.R., Alfano S., Wang Q., Ho P.P. (2001). Remote-Controllable, Micro-Scale Device for Use in in Vivo Medical Diagnosis and/or Treatment. U.S. Patent.

[151] Alfano R., Katz A., Alfano S. (2011). Micro-Scale Compact Device for in Vivo Medical Diagnosis Combining Optical Imaging and Point Fluorescence Spectroscopy. U.S. Patent.

[152] Gono K., Obi T., Yamaguchi M., Oyama N., Machida H., Sano Y., Yoshida S., Hamamoto Y., Endo T. (2004). Appearance of Enhanced Tissue Features in Narrow-Band Endoscopic Imaging. J. Biomed. Opt..

[153] Gono K. (2017). An Introduction to High-Resolution Endoscopy and Narrowband Imaging. Comprehensive Atlas of High-Resolution Endoscopy and Narrowband Imaging.

[154] Van den Broek F.J.C., Fockens P., Dekker E. (2007). Review Article: New Developments in Colonic Imaging. Aliment. Pharmacol. Ther..

[155] Machida Y., Hamamoto Y., Muto M., Kozu T., Tajiri H., Yoshida S. (2004). Narrow-Band Imaging in the Diagnosis of Colorectal Mucosal Lesions: A Pilot Study. Endoscopy.

[156] Muto M., Horimatsu T., Ezoe Y., Morita S., Miyamoto S. (2009). Improving Visualization Techniques by Narrow Band Imaging and Magnification Endoscopy. J. Gastroenterol. Hepatol..

[157] Kuznetsov R., Rey J.-F. (2006). Narrow-Band Imaging: Potential and Limitations. Endoscopy.

[158] Gono K., Yamazaki K., Doguchi N., Nonami T., Obi T., Yamaguchi M., Ohyama N., Machida H., Sano Y., Yoshida S. (2003). Endoscopic Observation of Tissue by Narrowband Illumination. Opt. Rev..

[159] Wong Kee Song L.M., Adler D.G., Conway J.D., Diehl D.L., Farraye F.A., Kantsevoy S.V., Kwon R., Mamula P., Rodriguez B., Shah R.J. (2008). Narrow Band Imaging and Multiband Imaging. Gastrointest. Endosc..

[160] Yao M., Fujisaki J. (2005). Techniques Using the Hemoglobin Index of the Gastric Mucosa. Endoscopy.

[161] Kato M., Nakagawa S., Shinmizu Y., Sugiyama T., Asaka M. (2002). The efficacy of magnifying endoscopy with adaptive index of hemoglobin enhancement for diagnosis of helicobacter pylori-induced gastritis. Dig. Endosc..

[162] Toyota Y., Honda H., Omoya T., Inayama K., Suzuki M., Kubo K., Nakasono M., Muguruma N., Okamura S., Shimizu I. (2002). Usefulness of a Hemoglobin Index Determined by Electronic Endoscopy in the Diagnosis of Helicobacter Pylori Gastritis. Dig. Endosc..

[163] Macleod H.A. (2017). Thin-Film Optical Filters.

[164] Correia J.H.G. (1999). Optical Microsystems in Silicon Based on a Fabry-Perot Resonance Cavity: Application for Spectral Analysis of Visible Light.

[165] Gounella R.H., Granado T.C., da Costa J.P.C., Carmo J.P. (2021). Optical Filters for Narrow Band Light Adaptation on Imaging Devices. IEEE J. Sel. Top. Quantum Electron..

[166] Ragunath K., Chiu P. (2022). A primer to image enhanced endoscopy. Transl. Gastroenterol. Hepatol..

[167] Akarsu C., Sahbaz N.A., Dural A.C., Unsal M.G., Kones O., Kocatas A., Halicioglu I., Alis H. (2016). FICE vs Narrow Band Imaging for In Vivo Histologic Diagnosis of Polyps. JSLS.

[168] Subramaniam S., Kandiah K., Schoon E., Aepli P., Hayee B., Pischel A., Stefanovic M., Alkandari A., Coron E., Omae M. (2020). Development and validation of the international Blue Light Imaging for Barrett’s Neoplasia Classification. Gastrointest. Endosc..

[169] Osawa H., Yamamoto H., Miura Y., Sasao W., Ino Y., Satoh H., Satoh K., Sugano K. (2014). Blue Laser Imaging Provides Excellent Endoscopic Images of Upper Gastrointestinal Lesions. Video, J. Encycl. GI Endosc..

[170] Lee C.K., Lee S.H., Hwangbo Y. (2011). Narrow-band imaging versus I-SCAN for the real-time histological prediction of diminutive colonic polyps: A prospective comparative study by using the simple unified endoscopic classification. Gastrointest. Endosc..

[171] Cammarota G., Ianiro G., Sparano L. (2013). Image-Enhanced Endoscopy with I-SCAN Technology for the Evaluation of Duodenal Villous Patterns. Dig. Dis. Sci..

